# A new subtype of diffuse midline glioma, H3 K27 and BRAF/FGFR1 co-altered: a clinico-radiological and histomolecular characterisation

**DOI:** 10.1007/s00401-023-02651-4

**Published:** 2023-12-08

**Authors:** Lucie Auffret, Yassine Ajlil, Arnault Tauziède-Espariat, Thomas Kergrohen, Chloé Puiseux, Laurent Riffaud, Pascale Blouin, Anne-Isabelle Bertozzi, Pierre Leblond, Klas Blomgren, Sébastien Froelich, Alberto Picca, Mehdi Touat, Marc Sanson, Kévin Beccaria, Thomas Blauwblomme, Volodia Dangouloff-Ros, Nathalie Boddaert, Pascale Varlet, Marie-Anne Debily, Jacques Grill, David Castel

**Affiliations:** 1 Molecular Predictors and New Targets in Oncology, Inserm, Gustave Roussy, Université Paris-Saclay, Villejuif, France; 2 Department of Neuropathology, GHU Paris-Psychiatrie et Neurosciences, Sainte-Anne Hospital, Paris, France; 3 UMR 1266, IMA-Brain, Institut de Psychiatrie et Neurosciences de Paris, Inserm, Paris, France; 4 Département de Cancérologie de L’Enfant et de L’Adolescent, Gustave Roussy, Université Paris-Saclay, Villejuif, France; 5 Department of Pediatric Oncology, Rennes University Hospital, Rennes, France; 6 Department of Pediatric Neurosurgery, Rennes University Hospital, Rennes, France; 7 Department of Pediatric Hematology, CHRU de Tours, Tours, France; 8 Department of Pediatric Hematology-Oncology, Hôpital des Enfants, Toulouse, France; 9 Institute of Pediatric Hematology and Oncology, Centre Léon Bérard, Lyon, France; 10 Department of Women’s and Children’s Health, Karolinska Institutet, Stockholm, Sweden; 11 Pediatric Hematology and Oncology, Karolinska University Hospital, Stockholm, Sweden; 12 Service de Neurochirurgie-Hôpital Lariboisière, Assistance Publique-Hôpitaux de Paris, Université Paris Cité, Paris, France; 13 Inserm U1127, CNRS UMR 7225, Institut du Cerveau, ICM, Charles Foix, Service de Neurologie 2-Mazarin, Sorbonne Université, AP-HP, Hôpitaux Universitaires la Pitié Salpêtrière, Paris, France; 14 Department of Pediatric Neurosurgery, Hôpital Necker-Enfants Malades, AP-HP, Université Paris Cité, Paris, France; 15 Department of Pediatric Radiology, Hôpital Necker-Enfants Malades, AP-HP, Université Paris-Cité, Paris, France; 16 INSERM U1163, Institut Imagine, Université Paris Cité, Paris, France; 17 Département de Biologie, Université Évry Paris-Saclay, Évry, France

**Keywords:** Paediatric-type high-grade glioma, Adult glioma, Midline glioma, BRAF-V600E mutation, FGFR1 mutation, DNA methylation profiling

## Abstract

*Diffuse midline gliomas (DMG) H3 K27-altered* are incurable grade 4 gliomas and represent a major challenge in neuro-oncology. This tumour type is now classified in four subtypes by the 2021 edition of the WHO Classification of the Central Nervous System (CNS) tumours. However, the H3.3-K27M subgroup still appears clinically and molecularly heterogeneous. Recent publications reported that rare patients presenting a co-occurrence of H3.3K27M with *BRAF* or *FGFR1* alterations tended to have a better prognosis. To better study the role of these co-driver alterations, we assembled a large paediatric and adult cohort of 29 tumours H3K27-altered with co-occurring activating mutation in *BRAF* or *FGFR1* as well as 31 previous cases from the literature. We performed a comprehensive histological, radiological, genomic, transcriptomic and DNA methylation analysis. Interestingly, unsupervised t-distributed Stochastic Neighbour Embedding (tSNE) analysis of DNA methylation profiles regrouped *BRAF*^*V600E*^ and all but one *FGFR1*^*MUT*^ DMG in a unique methylation cluster, distinct from the other DMG subgroups and also from ganglioglioma (GG) or high-grade astrocytoma with piloid features (HGAP). This new DMG subtype harbours atypical radiological and histopathological profiles with calcification and/or a solid tumour component both for *BRAF*^*V600E*^ and *FGFR1*^*MUT*^ cases. The analyses of a H3.3-K27M *BRAF*^*V600E*^ tumour at diagnosis and corresponding in vitro cellular model showed that mutation in *H3-3A* was the first event in the oncogenesis. Contrary to other DMG, these tumours occur more frequently in the thalamus (70% for *BRAF*^*V600E*^ and 58% for *FGFR1*^*MUT*^) and patients have a longer overall survival with a median above three years. In conclusion, *DMG, H3 K27 and BRAF/FGFR1 co-altered* represent a new subtype of DMG with distinct genotype/phenotype characteristics, which deserve further attention with respect to trial interpretation and patient management.

**Supplementary Information:**

The online version contains supplementary material available at 10.1007/s00401-023-02651-4.

## Introduction

Thanks to significant advances in genomics, the 2016 then-2021 World Health Organization (WHO) Classification of Tumours of the Central Nervous System (CNS) has defined tumour entities based on histological but also molecular features, like the driver genetic event [15, 16]. *Diffuse midline gliomas (DMG) H3 K27-altered* have now been identified as a new type of malignant gliomas which occur in the paediatric and adult populations, although with disparities according to the preferential location, *i.e.* brainstem in children and thalamus in adults [18, 45].

DMG H3 K27-altered are either characterised by the substitution in histone H3 of the lysine at position 27 by a methionine (H3K27M), or the overexpression of *EZHIP* [3, 42]. Both mechanisms lead to Polycomb Repressor Complex 2 (PRC2) inhibition with a global loss of H3K27me3 [5, 13], and consequently a major epigenetic and transcriptomic remodelling [1, 3]. According to different molecular and clinical parameters, including specific DNA methylation profiles, DMG H3 K27-altered appeared more heterogeneous than initially thought, and were further classified in four subtypes: *H3-3A* K27-mutant (H3.3-K27M), *H3C2* K27-mutant (H3.1-K27M), H3-wild-type (with *EZHIP* overexpression) or *EGFR*-altered [3, 4, 16, 19, 33]. Despite this subdivision, we still observed some clinical and molecular heterogeneity within the most common H3.3-K27M subtype. We previously demonstrated that *TP53* co-driver mutations are associated with a worse tumour response to radiotherapy and a poorer outcome [41] suggesting that additional molecular alterations can deeply modify the phenotype induced by the driver histone H3 mutation. Recently, we and others have described single cases or small studies of tumours with concomitant alterations of H3-K27M and mitogen-activated protein kinase (MAPK) pathway showing a possible longer survival compared to patients with DMG H3 K27-altered, *BRAF* and *FGFR1* wild-type. In these studies, diagnoses ranged from H3.3-K27M *BRAF*^*V600E*^/*FGFR1*^*MUT*^ pilocytic astrocytoma or midline gangliogliomas grade 1–3 [11, 21–23, 26, 29, 44] to diffuse gliomas grade 4 [25, 31]. As *BRAF* and *FGFR1* mutations are typical hallmarks of low-grade gliomas/glioneuronal tumours such as ganglioglioma or pilocytic astrocytoma, the co-occurrence of H3-K27 and these MAPK alterations makes diagnosis and grading difficult [30]. In order to understand how these alterations could mitigate the prognosis of these neoplasms, we analysed (radiologic, histologic, genomic, transcriptomic and DNA methylation analyses) a larger DMG H3 K27-altered cohort, comprising 29 tumours harbouring *BRAF*^*V600E*^ or *FGFR1*^*MUT*^ complemented by paediatric and adult cases from the literature.

## Patients, materials and methods

### Patients and tumour samples

The first part of the cohort is composed of 60 patients diagnosed with H3-K27M or EZHIP-overexpressing tumours harbouring a coding mutation in *BRAF* or *FGFR1* genes, from the Necker Enfants-Malades/GHU-Sainte Anne Hospital/Gustave Roussy center and Biomede 1 Trial (NCT02233049) (*n* = 29), or from other published cohorts (*n* = 31) [3, 18, 23, 25, 30, 31, 37]*.* The control cohort includes patients with wild-type *FGFR1/BRAF,* in the above-mentioned cohorts of H3K27M or *EZHIP-*overexpressing DMG (flowchart in Supplementary Fig. 1, online resource). Tumour tissue and clinical data were collected under informed consent obtained from the parents or guardian according to the IRB approved protocol (CNIL 1176643).

### Radiology analysis

All available radiology outcomes at diagnosis (CT and MRI) of patients with H3.3-K27M tumours from our cohort were reviewed centrally by three experts (VDR, NB, and JG). Parameters specifically recorded were: radiological presentation (diffuse, circumscribed or nodular and diffuse), presence of contrast enhancement (yes/no) and calcifications (yes/no). For circumscribed tumours, the pattern at evolution was also analysed.

### Histopathological analyses and immunohistochemistry

Formalin-fixed paraffin-embedded (FFPE) tissue samples for each patient were retrieved and Haematoxylin–Phloxine–Saffron (HPS)-stained slides were analysed by two experienced neuropathologists (PV and ATE) to confirm morphological diagnoses. Micro-calcifications were noted as present or absent as well as granular bodies, ganglion neurons, necrosis, and microvascular proliferation. Mitotic activity (per 2 mm^2^) and tumour growth architecture were analysed within the inherent limits of a stereotaxic biopsy exploration. The latter was labelled as diffuse, compact tumoral areas or both. Morphological aspects were evaluated as ganglioglioma-like (GG-like), HGG with piloid astrocytic component or DMG-like. The infiltration pattern was also assessed by NF70 immunostaining (*i.e.* residual NF70 network or not). Immunostaining was performed from 3 μm-thick representative FFPE sections using a Dako OMNIS automate. The following primary antibodies were used: H3K27me3 (1:2500, polyclonal, Diagenode), H3-K27M (1:5000, clone EPR18340, Abcam), Neurofilament Protein NF70 (1:100, clone 2F11), CD34 (1:40, clone QBEnd-10, Dako), BRAFV600E (1:100, clone VE1, Abcam), ATRX (1:200, polyclonal, Diagomics), Ki-67 (1:200, clone MIB-1, Dako). Antigen retrieval was performed at 95 °C, pH9 (GV80011-2, Dako) or pH6 (GV805, Dako). External positive and negative controls were used for antibody validation.

### DNA/RNA extraction and sequencing

DNA and RNA were extracted from frozen tumours using Allprep DNA/RNA kit (Qiagen) and were quantified using, respectively, the Qubit Broad Range double-stranded DNA assay (Life Technologies) or the Qubit RNA high sensibility (Life Technologies). When no frozen material was available for DNA methylation profiling, tumour DNA was extracted from formol-fixed paraffin-embedded (FFPE) block sections using dedicated protocols at Diagenode or Integragen. Targeted DNA sequencing (≥ 6000× coverage) or whole exome sequencing (≥ 130× coverage) was performed as previously described [10, 41]. WES was aligned with BWA according to GATK best practice guidelines, then Mutect2 was used for the DNA calling. RNAseq on DMG primary tumours was performed at Integragen (Evry, France). PolyA mRNA molecules were initially purified from at least 100 ng total RNA (NEBNext® Poly(A) mRNA Magnetic Isolation Module, NEB) and libraries were then prepared using the NEBNext Ultra II Directional RNA Library Prep Kit (NEB). Paired-end reads of 100 bp were generated on an Illumina NovaSeq reaching an average sequencing depth of 60 million reads.

### DNA Methylation array processing

Genome-wide DNA methylation analysis was performed using either the Illumina HumanMethylation450 BeadChip (450 k) or EPIC arrays as previously published [2, 3]. Data were obtained from different platforms (DKFZ Heidelberg; Integragen; Diagenode; published data) and were analysed with R (v4.0.4). For t-Distributed Stochastic Neighbour Embedding (t-SNE) analysis, the *minfi* package was used to load idat files and preprocessed with the function *preprocess.illumina* for dye bias and background correction. Probes located on sex chromosomes or not uniquely mapped to the human reference genome were removed. Probes containing single-nucleotide polymorphisms or that were not present in both EPIC and 450 k methylation array were also eliminated. A batch effect correction was done with *removebatchEffect* function from *limma* package, to remove difference between formalin-fixed paraffin-embedded and frozen samples. The probes were sorted by standard deviation. The 10,000 most variable probes were used for subsequent clustering analysis and to compute the 1-variance weighted Pearson correlation between samples. The distance matrix was used as input in t-SNE from *Rtsne* package. For the second analysis, DNA methylation-based classification of CNS tumours from DKFZ-Heidelberg was used in order to predict the CNS tumour class based on the V12.7 of the classifier (www.molecularneuropathology.org).

### Gene expression analysis

Reads were pre-processed using the nf-core RNAseq pipeline (v3.0), mapped to the reference genome GRC38/hg38 with the *STAR* tool (v2.6.1d), annotated with GENCODE v36 and counting was performed with the Salmon quantification tool (v1.4.0). Differential gene expression analysis was performed with the *DESeq2* package (v1.30.0, *minReplicatesForReplace* = 7, *betaPrior* = TRUE) with a threshold of 0.01 for Benjamini–Hochberg adjusted p value (adj-p). For gene set enrichment analysis (GSEA), hypergeometric tests were used to identify overrepresented gene sets from the *MSigDB* v7.4 database, amongst genes ranked by significance and fold-change in differential expression analysis, with Benjamini–Hochberg multiple testing correction using the package *Clusterprofiler*. Catalogues considered included Hallmark and C2. Differences were considered as significant when false discovery rate adj-*q* value was < 0.02. Gene expression comparison was evaluated with Wald test using *DESeq2*.

### Univariate and multivariate survival analyses

Overall survival (OS) was estimated with the Kaplan–Meier method and median overall survival was computed using a log-rank test. OS was obtained from the post-diagnosis until death patient or last known information. The analysis was realised in Prism9 software. Multivariable Cox proportional hazards regression model on OS was performed including histone H3, *BRAF*, *FGFR1*, *TP53* status, age at diagnosis and tumour location with R software using the function *coxph()* of the *survival package* (Version 3.2–13).

### Statistical analyses

Distribution of age at diagnosis according to different parameters was accessed by Mann–Whitney test. Presence of macro-calcification, contrast enhancement, radiologic profile and sex ratio were evaluated by Fisher’s exact test. Chi-square test for trend was used to evaluate tumour type and location. All statistic tests were performed using Prism 9 software (GraphPad).

## Results

### Genomic landscape of DMG H3K27-altered with *BRAF/ FGFR1* alterations

**Fig. 1:**
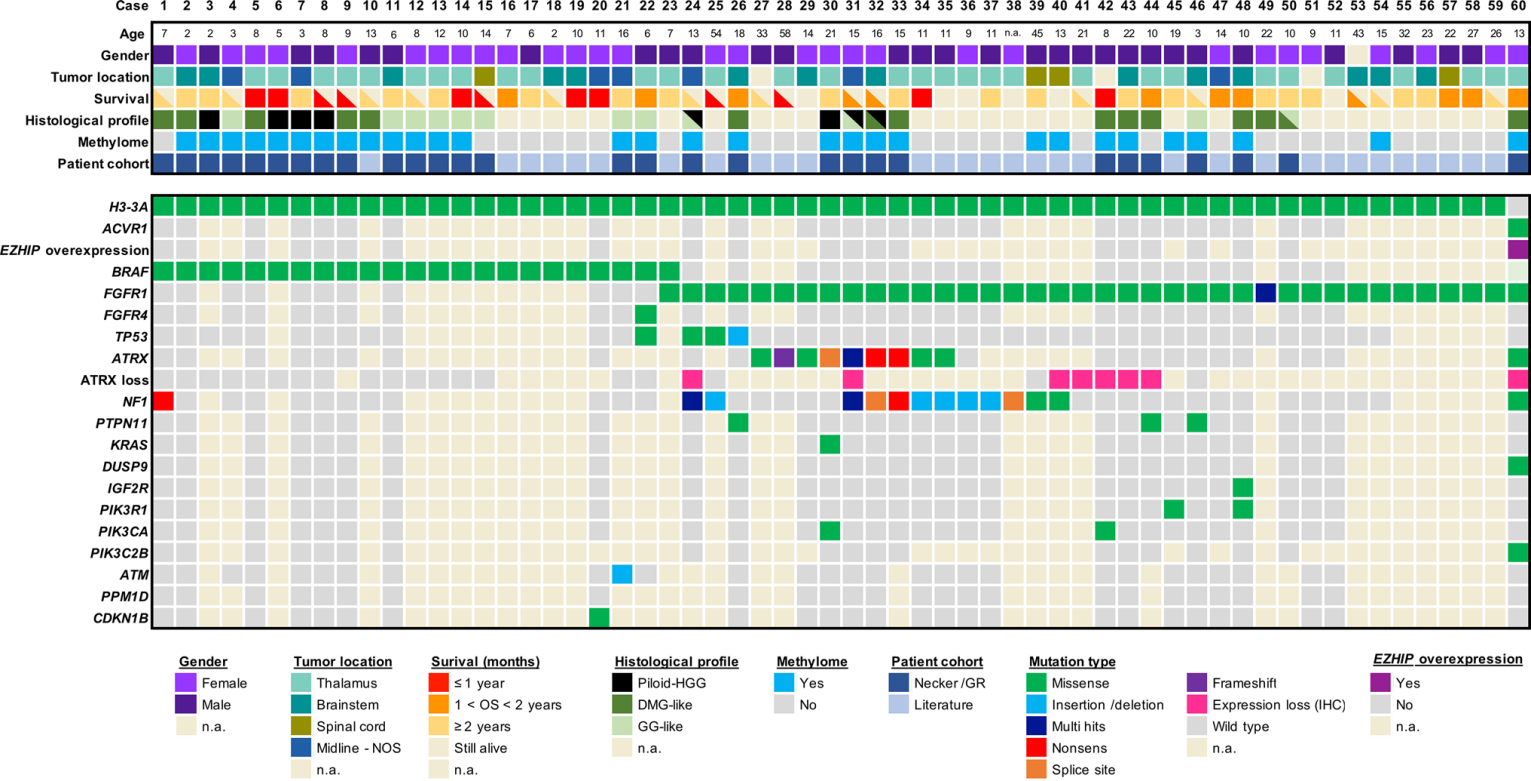
Clinical and molecular characteristics of the patient cohort of DMG H3-K27M with *BRAF and FGFR1* mutations. Overview of the clinical and molecular annotations of 60 paediatric and adult DMG H3-K27 patients presenting *BRAF* or *FGFR1* mutations. Cases are presented in columns and genes status in rows. Age is reported in years. All molecular information is derived from DNA or RNA sequencing analyses, except immuno-histological data (including histological profile, EZHIP and ATRX expression). For survival, patients still alive at last follow-up are indicated by a half-filled square

We analysed a study cohort of H3K27-altered gliomas harbouring mutations in *BRAF* (*n* = 22), *FGFR* (*n* = 37), or both (*n* = 1; Supplementary Fig. 1 and Table 1, online resource). Of these*,* 43 were children (< 18 years) and 16 adults. In parallel, we analysed control cases of patients with *BRAF*^*WT*^*/FGFR*^*WT*^ DMG H3-K27 from the corresponding cohorts. All DMG H3-K27M with *BRAF* alterations harboured a somatic V600E substitution, but no fusion (Fig. 1). Concerning *FGFR*, all cases displayed *FGFR1* hotspot substitutions N546K/D (73%, 27/37) and/or K656E/M (23%, 11/37) preferentially occurring in CNS tumours, but neither *FGFR1* fusion/duplication nor FGFR2/3 alterations (Fig. 1) [20]. All these mutations in *BRAF* and *FGFR1* are widely known to induce an aberrant MAPK activation [12, 20, 27, 38, 43]. We estimated the overall prevalence of DMG *BRAF*^*V600E*^ and DMG *FGFR1*^*MUT*^ within the H3.3-K27M tumours in our composite paediatric–adult cohort at 7.6% and 12.3%, respectively. Thus, DMG *BRAF*^*MUT*^*/FGFR1*^*MUT*^ together could represent close to 20% of all DMG H3.3-K27M.

**Table 1:** Multivariable Cox proportional hazards regression model for the OS of patients with H3-K27M DMG

Variable	Hazard ratio (95%CI)	*p* value
H3 status		
H3.1-K27M	1	
H3.3-K27M	1.5820 (1.0529–2.3769)	0.0274
H3-WT	1.0210 (0.5452–1.9119)	0.9482
*BRAF* status		
* BRAF* wild-type	1	
* BRAF* mutated	0.2132 (0.1098–0.4140)	5.03e-06
*FGFR1* status		
* FGFR1* wild-type	1	
* FGFR1* mutated	0.3414 (0.1963–0.5939)	0.0001
*TP53* status		
* TP53* wild-type	1	
* TP53* mutated	1.4622 (1.0601–2.0169)	0.0205
Age at diagnosis	0.9582 (0.9361–0.9809)	0.0003
Tumour location		
Pons	1	
Thalamus	0.7084 (0.4802–1.0452)	0.0823
Other midline	1.0039 (0.6263–1.6089)	0.9872

*CI* confidence interval, *WT* wild-type

We further analysed the genomic landscape of tumour for which material or data were available and observed that *BRAF*^*MUT*^ or *FGFR1*^MUT^ was mostly associated with H3.3-K27M mutation but not H3.1-K27M mutation. One tumour harboured a *FGFR1*^*MUT*^ in the context of a DMG H3-K27 wild-type with *EZHIP* overexpression presenting an *ACVR1* mutation (case #60). Second, *BRAF* and *FGFR1* mutations were mutually exclusive, as only one tumour presented both hits (case #23; Fig. 1). However, no clonality information was available for this case to confirm subclonality of the two mutations. Finally, we observed that in 90% (9/10) of *FGFR1*^*MUT*^ cases for which we had the information, *FGFR1* mutation was clonal to *H3-3A* mutation with a similar variant allele frequency suggesting a role in the early steps of oncogenesis of these tumours. It was less frequently the case for patients with DMG H3-K27M *BRAF*^*V600E*^ with only 33% (2/6) tumours where *BRAF* mutation appeared clonal to H3-K27M mutation. In addition, H3-K27 *FGFR1*^*MUT*^ tumours presented often other hits in the MAPK pathway with *NF1* (13/31; 42%) or *PTPN11* in (3/22; 13.6%) as the topmost mutated genes (Fig. 1). Additional MAPK-activating mutations seemed to be less frequent for *BRAF*^*MUT*^ tumours with 1/12 (8.3%) case harbouring an *NF1* mutation. *TP53* mutations were found in 5% (1/20) and 9.3% (3/32) of the *BRAF*^*MUT*^ and *FGFR1*^*MUT*^ tumours, respectively but not *PPM1D* (Fig. 1). For one H3.3-K27M *FGFR1*^*MUT*^ patient, *TP53* mutation was clonal to H3.3-K27M and it was sub-clonal for the other case for which information was available. In addition, *ATRX* was mutated, or its expression was lost on IHC in 59.3% (16/27) of *FGFR1*^*MUT*^ DMG, albeit never in *BRAF*^*MUT*^ tumours, and this was not associated to *TP53*^*MUT*^ in all but one case (#24). A mutation in a member of the PI3K/AKT/mTOR signalling pathway was present in 19% (4/21) of H3-K27M-*FGFR1*^*MUT*^ tumours (Fig. 1).

### *BRAF*^*MUT*^*/FGFR1*^*MUT*^ H3 K27-altered tumours are histologically heterogeneous with frequent mixed diffuse-circumscribed presentation and calcifications

**Fig. 2:**
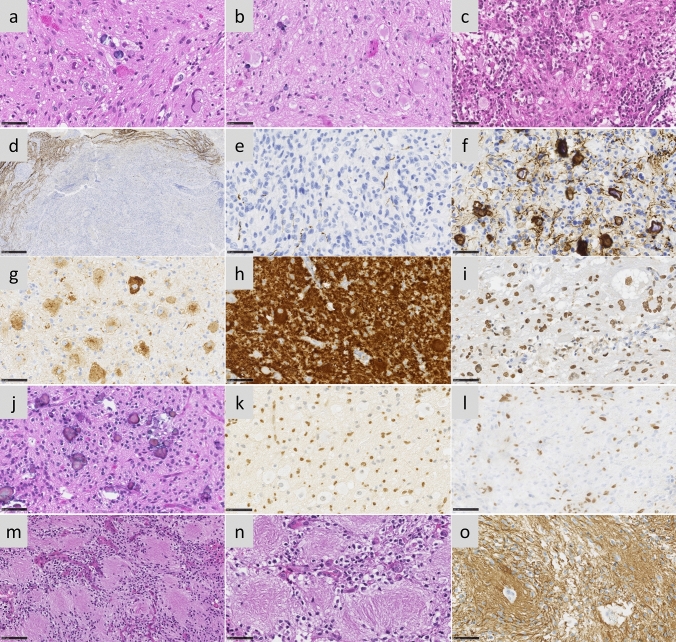
Multiple histopathological profiles of DMG H3-K27 with *BRAF or FGFR1* mutations. Case 14 **a** A glioneuronal proliferation with ganglion cells, eosinophilic granular bodies and some microcalcifications (HPS, magnification × 400). Case 11 **b** A glioneuronal proliferation with numerous ganglion cells (HPS, magnification × 400). Case 13 **c** A glioneuronal proliferation with numerous ganglion cells and lymphocytic infiltrates (HPS, magnification × 400). Case 31 **d** A mainly circumscribed proliferation (neurofilament, magnification × 30). Case 32 **e** A mainly circumscribed proliferation (neurofilament, magnification × 400) with a diffuse component at the periphery of the tumour **f** (neurofilament, magnification × 400). **g** Diffuse chromogranin A immunoreactivity staining neuron cells (magnification × 400). **h** BRAF^V600E^ expression in all tumour cells including ganglion cells (magnification × 400). **i** H3K27M expression in all tumour cells including ganglion cells (magnification × 400). Case 7 **j** A glial proliferation with oligo-like features and microcalcifications (magnification × 400). Case 11 **k** Global loss of H3K27me3 (magnification × 400). (**l**) Loss of ATRX in tumour cells (magnification × 400). **m** Whorls of gliofibrillary processes (HPS, magnification × 20). **n** Whorls of gliofibrillary processes (HPS, magnification × 400), stained using GFAP antibody **o**, magnification × 400). Black scale bars represent 50 μm (**a**–**c**, **e**–**l** and **n**–**o**), 100 µm (**m**), and 500 µm (**d**)

We next analysed the histo-pathological profile of cases of our own cohort (Supplementary Table 1). These 29 tumours were initially diagnosed as DMG H3K27 (*n* = 13), ganglioglioma grade 1 (*n* = 5), anaplastic ganglioglioma grade 3 (*n* = 4) or other gliomas (GBM *n* = 3; pilocytic astrocytoma *n* = 1; LGG *n* = 1 or oligo-astrocytoma grade 3 *n* = 2). All *BRAF*^*MUT*^*/FGFR1*^*MUT*^ DMG presented a H3K27 trimethylation loss as expected and were positive for H3K27M staining with the exception of the case #60, harbouring *EZHIP* overexpression. Tumour growth pattern evaluated morphologically and based on NF70 immunostaining revealed that 12 tumours were only diffuse (*n* = 3 in *FGFR1*^*MUT*^ group and *n* = 9 in *BRAF*^*MUT*^), 4 were only composed of compact tumoral tissue and 13 were mixed diffuse with nodular compact areas. We observed three major histological patterns: GG-like in 41.3% (12/29), DMG-like in 34.4% (10/29), and HGG with a piloid astrocytic component (piloid-HGG) in 24.3% (7/29). *BRAF*^*MUT*^ and *FGFR1*^*MUT*^ tumours presented a classic DMG histological profile only in 25% (4/16) and 46% (6/13) of cases, respectively (Figs. 1, 2, Supplementary Fig. 2a, online resource). A GG-like profile, characterised by mixed neuronal and glial tumour cells, was present in 50% (8/16) and 30% (4/13) of *BRAF*^*MUT*^ or *FGFR1*^*MUT*^ cases, respectively (Fig. 2b–i). Finally, 25% (4/16) of *BRAF*^*MUT*^ and 23% (3/13) of *FGFR1*^*MUT*^ DMG presented a piloid-HGG profile with piloid cell morphology (Fig. 2j, k). The remaining *FGFR1*^*MUT*^ DMG case (3/13) harboured a mixed DMG-like and GG-like or piloid-HGG, and the last GG-like and piloid-HGG profile (Fig. 2l). Micro-calcifications, albeit rare in classic DMG, were observed in 56% (9/16) of *BRAF*^*MUT*^ and 38% (5/13) *FGFR1*^*MUT*^ DMG (Fig. 2j). Ganglion cells and eosinophilic granular bodies were seen in 37% (11/29) and 31% (9/29) mainly in the *BRAF*^*MUT*^ group (7/16) (Fig. 2b). GFAP whorls defined as a small gliofilament tangle, represented a rare but particular aspect (*n* = 4) (Fig. 2m–o). Perivascular lymphoid infiltrates were frequent (48%, 14/29) and especially enriched in the *BRAF*^*MUT*^ group (62%, 10/16) (Fig. 2c). CD34 extravascular staining was observed in the majority of tested cases: 50% (4/8) in *FGFR1*^*MUT*^ and 60% (9/15) in *BRAF*^*MUT*^. ATRX loss of expression was frequently seen in *FGFR1*^*MUT*^ cases tested (8/11) but absent in *BRAF*^*MUT*^ (0/16) (Figs. 1, 2l). The histopathological profile of *BRAF*^*MUT*^ or *FGFR1*^*MUT*^ DMG thus appeared very heterogeneous and distinct compared to classical DMG H3-K27M.

**Fig. 3:**
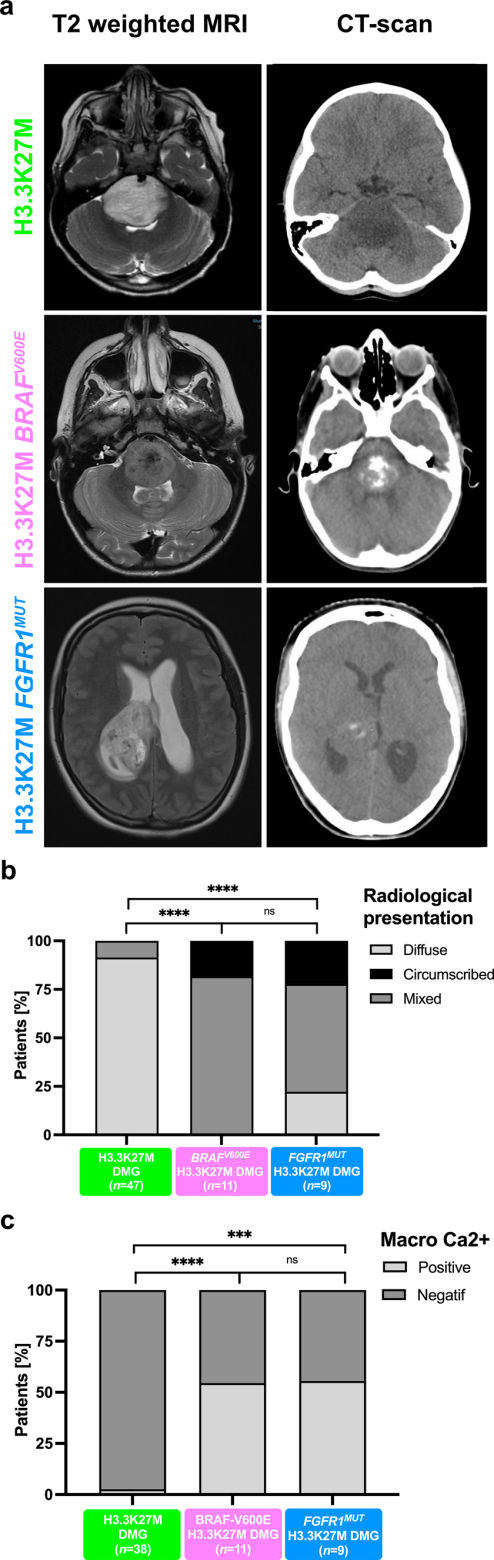
Radiological specificities of DMG H3-K27M *BRAF*^*V600E*^* or FGFR1*^*MUT*^. **a** T2-FLAIR (Fluid-attenuated inversion recovery) MR images sequences or CT scans (computed tomography) of DMG H3-K27M according to *BRAF* or *FGFR1* mutation status. **b** Comparison of the tumour radiological presentation (diffuse, circumscribed, or mixed) of DMG according to their genotype (chi-square test for trend: ns, ****p value < 0.0001). **c** Comparison of presence of macro-calcifications in DMG CT-scans according to the presence of MAPK alteration (Fisher’s exact test: ****p value < 0.0001, *p = 0.0210, *p = 0.0348)

Radiologically, we observed that DMG H3-K27 *BRAF*^*MUT*^ or *FGFR1*^*MUT*^ were mostly diffuse but presented a significant enrichment of a mixed nodular-diffuse aspect compared to control DMG H3-K27 (Fig. 3a–b; (9/11) 82% and (5/9) 56% *versus* (4/47) 8%; *p* value =  < 0.0001, chi-square test for trend). Few tumours were completely circumscribed (Fig. 3a, b—(2/11) 18% and (2/9) 22% versus (0/47) 0%; *p* value =  < 0.0001, Chi-square test for trend), and radiological analysis at progression identified a diffuse evolution pattern in half (2/4). Second, these tumours were more often contrast enhancing (Supplementary Fig. 2b, online resource; (11/11) 100% and (9/9) 100% *versus* 47% (22/47);* p* value = *0.0013 and 0.0029*, respectively; Fisher’s exact test*)*. Third, they developed more calcifications than DMG H3-K27 (Fig. 3a, c; (6/11) 55% and (5/9) 56% *versus* (1/38) 3%; *p* value =  < 0.0001, Fisher’s exact test). Presence of micro-calcifications detected in histological analyses correlated with macro-calcifications seen by CT scan in 4/8 (50%) DMG *BRAF*^*MUT*^ and 2/5 (33.3%) *FGFR1*^*MUT*^ (Supplementary Fig. 3c, d, online resource). In total in histologic and radiologic analyses, DMG H3K27M *BRAF*^*MUT*^ or *FGFR1*^*MUT*^ was calcified in 8/11 (72.7%) and 6/9 (66.7%), respectively. To evaluate how radiological features could detect DMG with MAPK alterations, we classified tumours according to a ‘classic’ (diffuse) or ‘atypical’ (nodular-diffuse or circumscribed aspect and/or presence of macro-calcifications) radiological profile independently of their genotype. This showed that 91% (10/11) of DMG with *BRAF*^*V600E*^ and 78% (7/9) DMG with *FGFR1*^*MUT*^ H3K27M DMG are classified as atypical *versus* only 8.5% (4/47) classical DMG H3K27 (Supplementary Fig. 3e, online resource;* p* value =  < 0.0001, Fisher’s exact test).

### DNA methylation profiling distinguishes a subgroup of *DMG H3 K27-altered* with MAPK-activating mutations

**Fig. 4:**
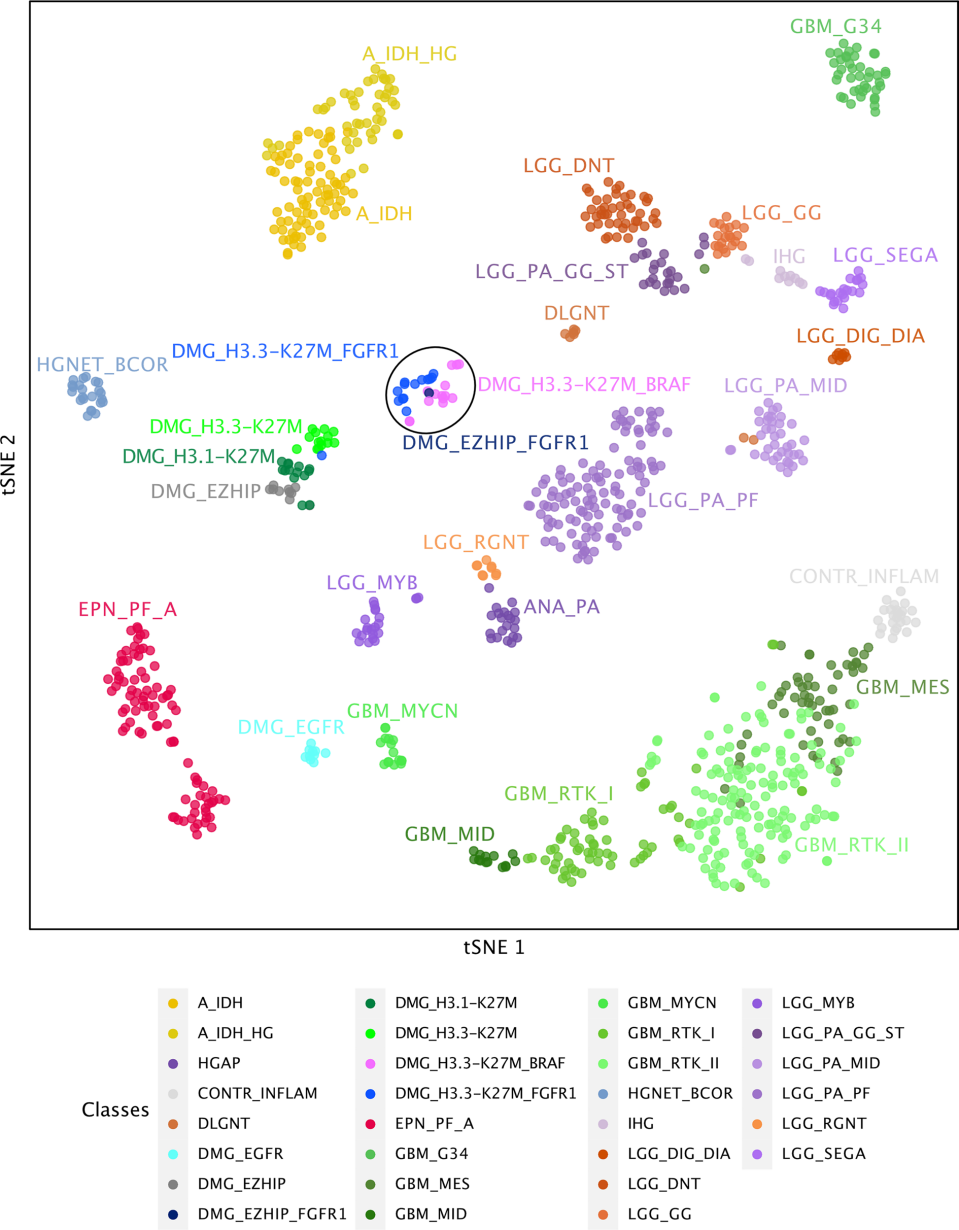
Analysis of DNA methylation profiles of DMG H3.3-K27M *BRAF*^*MUT*^*/FGFR1*^*MUT*^. CNS tumour classification based on DNA methylation profiles. Unsupervised clustering by t-SNE analysis of tumours based on their DNA methylation profiles using 10,000 topmost differentially methylated probes across the reference sample set composed of samples from *Capper *et al*.* (*n* = 936) and *Castel *et al*.* (*n* = 41)

Given the disparities between DMG_K27-BRAF/FGFR1 and classical DMG H3K27-altered, we hypothesised that DMG H3 K27-altered with MAPK-activating mutations might correspond to either (i) a new subtype of DMG or (ii) atypical aggressive MAPK-driven low-grade gliomas/glioneuronal tumours. To test these hypotheses, we analysed the DNA methylation profile of the whole cohort. Based on the Heidelberg DNA methylation brain classifier V12.8, 54% (7/13) of *BRAF*^*MUT*^ and 67% (10/15) of *FGFR*^*MUT*^ tumours classified as DMG_K27 and the remaining corresponded to other classes or were undefined (score < 0.9) (Supplementary Fig. 3a, online resource), which was consistent with the hypothesis that DMG H3 K27-altered with MAPK-activating mutation constitute a unique subtype of DMG. In contrast, all DMG H3.3-K27M *BRAF*^*WT*^*/FGFR*^*WT*^ clustered as DMG_K27. We then performed an unsupervised clustering based on DNA methylation profiles of these samples together with reference gliomas from the literature [2]. All DMG H3-K27 *BRAF*^*MUT*^ or *FGFR1*^*MUT*^ but one (case #42) separated from DMG H3-K27 on the tSNE, independently of their H3 mutational status or brain classifier score (Fig. 4, and supplementary Fig. 3b, online resource). They also separated from midline *BRAF*^*V600E*^ LGG and GG grade 1 (Supplementary Fig. 3c, online resource). Furthermore, they were grouped in subclasses according to the nature of the secondary MAPK mutation in *BRAF* or *FGFR1* (Fig. 4, and supplementary Fig. 3b, online resource).

### *BRAF* and *FGFR1* mutational status are prognostic in paediatric and adult *DMG H3 K27-altered*

**Fig. 5:**
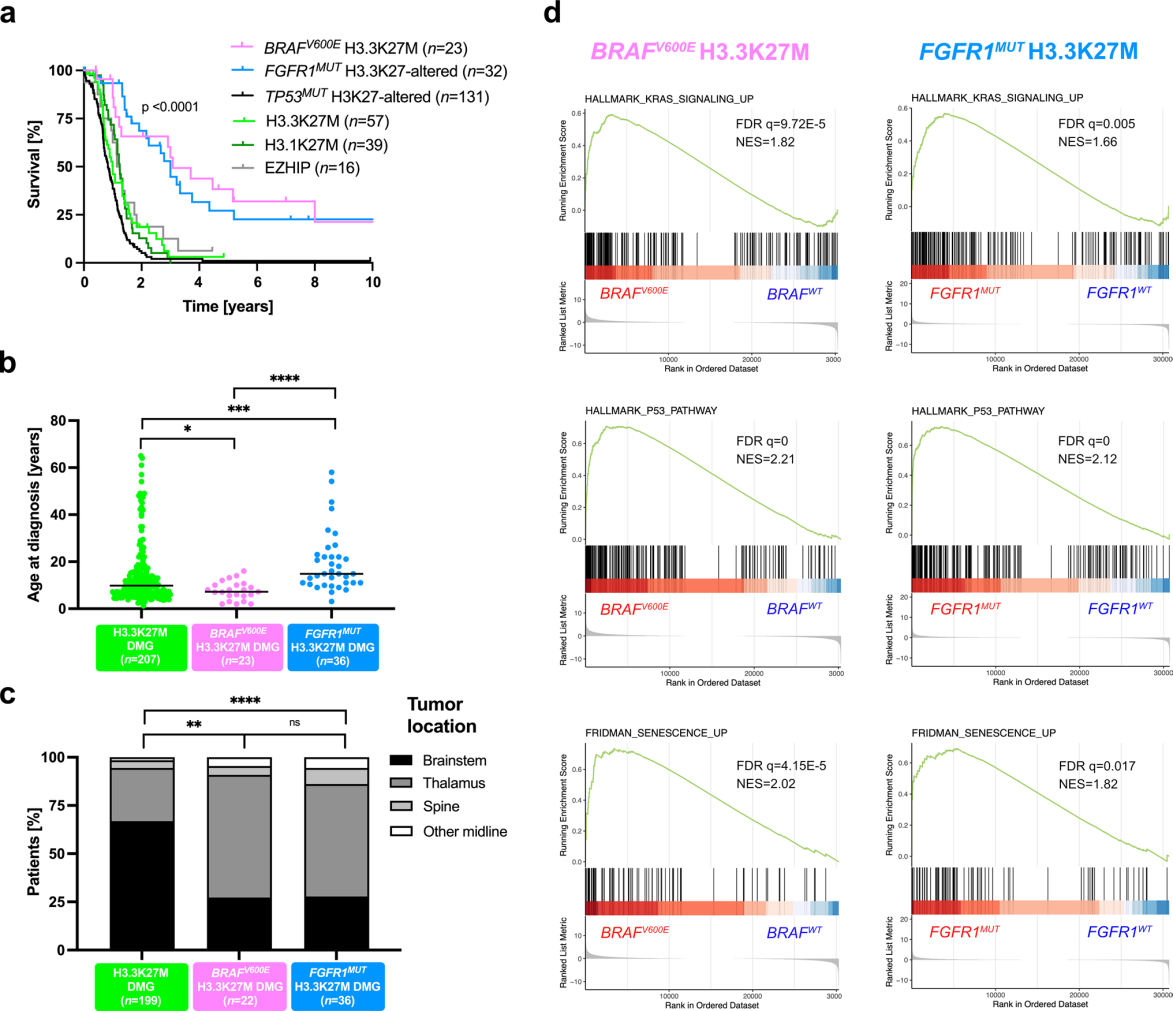
Clinical specificities of patients with DMG H3-K27M *BRAF*^*MUT*^*/FGFR1*^*MUT*^ and transcriptomic tumour profiling. **a** Comparison of OS estimated using Kaplan–Meier method according to the mutation status of Histone H3, *BRAF*, *FGFR1* and *TP53* (log-rank test, *p* value =  < 0.0001). **b** Distribution of age at diagnosis according to *BRAF* and *FGFR1* mutation status (Mann–Whitney test; *p value = 0.0168, ***p value = 0.0001, ****p value =  < 0.0001). **c** Comparison of tumour location according to the mutation status of *BRAF* and *FGFR1* (chi-square test for trend: ns, ***p value = 0.0004 ****p value =  < 0.0001). **d** GSEA plot showing common transcriptomic signature from DMG H3.3-K27M *vs.* DMG H3.3-K27M *BRAF*^*V600E*^ or *FGFR1*^*MUT*^. The normalised enrichment score (NES) and the false discovery rate (FDR q) are indicated in each plot

*TP53* mutations and *BRAF*^*V600E*^/*FGFR1* mutations are mostly mutually exclusive in DMG. In order to avoid the confounding effect of *TP53* mutations on the outcome of H3.3-K27M DMG without MAPK-activating alterations, we stratified patients on *Histone* H3 and *TP53* genotypes of both subgroups in Kaplan–Meier overall survival (OS) analyses. This analysis showed a significant better OS for paediatric and adult DMG H3-K27 patients with activating *BRAF* (median OS 37 mo.) or *FGFR1* mutations (median OS 36 mo.) compared to DMG H3.3-K27M *TP53*^*WT*^ (median OS 12 mo.) and other DMG subtypes (Fig. 5a; *p* value < 0.0001, global log-rank test). Further, we showed that there was no impact of histopathological features such as microvascular proliferation, necrosis or mitotic index, on the OS of patients with *BRAF* or *FGFR1-mutated* DMG (supplemental Fig. 2f-h, online resource). To pursue, we performed a multivariable analysis to evaluate the association of Histone H3, *BRAF*, *FGFR1* and *TP53* mutational status, age at diagnosis and tumoral location with survival. As expected from previous publications, histone H3 and *TP53* status were significantly associated with OS (Table 1) [4, 41]. Age was significantly associated with prognosis, but its overall impact was only marginal compared to other variables. We also showed that *BRAF*^*V600E*^ (HR: 0.2132, 95% CI 0.1098–0.4140, *p* value = 5.03e-06) and *FGFR1*^*MUT*^ (0.3414, 95%CI 0.1963–0.5939, *p* value = 0.0001) are strong and independent prognostic markers in H3K27-altered DMG (Table 1).

### Clinical disparities in paediatric and adult patients with DMG H3-K27M *BRAF/FGFR1*-mutated

We next compared other clinical parameters. The sex ratio was balanced in DMG with MAPK-activating mutations (Supplementary Fig. 4a, online resource). However, we identified a significant difference in age at diagnosis between H3.3-K27M DMG, *BRAF*^*MUT*^ and *FGFR1*^*MUT*^ DMG (Fig. 5b). More precisely, H3.3-K27M *BRAF*^*V600E*^ DMG developed only in children (< 18 years) with a median age at onset of 7.2 years lower than DMG H3.3K27M with 9.85 years (Fig. 5b; *p* value = 0.0123, Mann–Whitney test) but similar to DMG H3K27-altered paediatric cases with 7.6 years in a restricted paediatric cohort (supplementary Fig. 4b, online resource). In contrast, patients with *FGFR1*^*MUT*^ have a significant higher age at diagnosis compared to those *FGFR1*^*WT*^ with median of 14.8 and 9.8 years respectively (Fig. 5b; *p* value = 0.0001, Mann–Whitney test). The age at diagnosis of H3-K27M DMG can vary according to initial tumour locations, with a higher age of onset for thalamic *versus* pontine tumours [18, 34]. We found this difference of age according to tumour location in DMG H3.3-K27M and in DMG *BRAF*^V600E^ but not in DMG *FGFR1*^*MUT*^ (Supplementary Fig. 4c, online resource). Finally, DMG with *BRAF* and *FGFR1* mutations were significantly more frequent in the thalamus compared to DMG H3.3-K27M (Fig. 5c; 70% (16/23) and 58% (21/36), respectively *versus* 28% (55/199); *p* value = 0.0004, and *p* value < 0.0001, Chi-square test for trend). No variation in term of OS, age at diagnosis and tumour location was noted according to *FGFR1-mutated* variant: *FGFR1*^*N546K/D*^* versus FGFR1*^*N656E*^ (Supplementary Fig. 5, online resource).

### H3.3-K27M mutation occurs prior to *BRAF*^*V600E*^ during oncogenesis

We next wondered if the sequence of appearance of mutations was identical in these tumours, and more largely if they correspond to (i) DMG (H3-K27M as a first hit) or (ii) atypical aggressive low-grade gliomas (*BRAF*^*V600E*^ as first hit). To address this question, we analysed *BRAF* copy number variation (CNV) by digital droplet Polymerase Chain Reaction (ddPCR) on genomic DNA from one *BRAF*^*V600E*^ H3.3-K27M tumour (case #2) and from a H3.3-K27M *BRAF*^*WT*^ clone derived from this primary tumour in vitro (Supplementary Fig. 6, online resource). The *BRAF*^*WT*^ clone had two *BRAF* alleles and thus resulted from the re-amplification of an ancestral H3.3-K27M-only clone, but not from a genetic loss of the *BRAF*^*V600E*^ allele, demonstrating that, in one DMG H3.3-K27M *BRAF*^*V600E*^, H3.3-K27M was the first hit. Interestingly, DNA methylation profiles from this in vitro amplified *BRAF*^*WT*^ H3.3-K27M ancestral clone from case #2 and also from a tumour relapse enriched in H3.3-K27M *BRAF*^*WT*^ clone from patient #7 (initially diagnosed with DMG H3.3-K27M *BRAF*^*V600E*^), clustered with DMG H3-K27 with MAPK alterations instead of classical DMG H3-K27 (Supplementary Fig. 3b, online resource).

### DMG H3.3K27M with MAPK alterations show a transcriptomic signature of senescence with up-regulation of *CDKN1A* (P21)

In order to investigate the possible specificities of the DMG H3.3-K27M with *BRAF*^*MUT*^* or FGFR1*^*MUT*^, we compared their transcriptome to regular DMG H3.3-K27M *TP53*^*WT*^ separately. We found 676 significantly up-regulated and 633 down-regulated genes in the contrast H3.3K27M *BRAF*^*WT*^* versus* H3.3-K27M *BRAF*^*V600E*^ DMG and 228 up-regulated and 274 down-regulated genes in the contrast H3.3-K27M *FGFR1*^*WT*^* versus* H3.3-K27M *FGFR1*^*MUT*^ DMG (adj. *p* value ≤ 0.001) (Supplementary Fig. 7a, b, online resource). Ninety-four up-regulated and 111 down-regulated genes were common to the two comparisons. Using gene set enrichment analyses (GSEA), we observed an enrichment for MAPK signalling and PI3K/AKT/MTOR signalling signatures in both *BRAF*^*MUT*^ and *FGFR1*^*MUT*^ DMG (Fig. 5d; Supplementary Fig. 7e, f, online resource) as well as angiogenesis and hypoxia signatures (Supplementary Fig. 7 g-j, online resource). In addition, transcriptomic signatures highlighted activation of senescence and P53 signalling pathway in both comparisons (Fig. 5d). P53 protein is a tumour suppressor implicated in the permanent cell cycle arrest by inducing senescence or apoptosis in response to stress like oncogene activation [6, 14]. TP53 pathway activation was validated at the protein level by immunohistochemistry (IHC) in 71% (10/14) of DMG H3.3-K27M *TP53*^*WT*^ with *BRAF/FGFR1* alterations, showing heterogeneous, weak to strong TP53 staining. Moreover *CDKN1A*, encoding the senescence marker P21, was overexpressed at the RNA level in both *BRAF*^*MUT*^ and *FGFR1*^*MUT*^ gliomas compared to those only H3.3-K27M mutated (adj. *p* value < 0.0001) (Supplementary Fig. 7 k online resource). *CDKN2A*, which encodes the tumour suppressor P16, was overexpressed only in *BRAF*-mutant gliomas (Supplementary Fig. 7 l online resource; adj. *p* value = 0.0048). As a whole, based on transcriptome and IHC, DMG H3-K27 with *BRAF*^*MUT*^*/FGFR1*^*MUT*^ were characterised by a senescence programme, likely induced trough a P16/P21-P53 axis.

## Discussion

The recent description of ‘*DMG, H3 K27-altered’* and its sub-classification into four molecular subgroups does not capture completely the diversity of this disease [16]. Our data support the individualization of an additional new subtype of DMG with distinct histological, radiological, clinical, genomic, transcriptomic and epigenetic features that we provisionally termed *DMG, H3 K27 and BRAF/FGFR1 co-altered* (DMG_K27-BRAF/FGFR1) which may represent 20% of *DMG H3 K27-altered*. Using unsupervised analysis of DNA methylation tumour profiles, DMG_K27-BRAF/FGFR1 formed a specific cluster, separated from other DMG_K27 gliomas, others adult/paediatric diffuse gliomas, low-grade glial/glioneuronal tumours and more specifically *BRAF*^*V600E*^-mutated ganglioglioma even midline located.

This highlights a possible distinct cell of origin for DMG_K27- BRAF/FGFR1, able to exhibit a mixed glial and neuronal differentiation, mostly noticeable in the BRAF subclass [2, 39]. Schüller et al*.* did not mention any of these phenotypes in DMG H3-K27M *FGFR1*^*MUT*^, due to the limited number of tumours [31]. The analysis of the H3.3-K27M ancestral clone derived from a DMG *BRAF*^*V600E*^ H3.3K27M harboured this same DNA methylation profile, confirming that the specific DNA methylation profile of DMG_K27-BRAF/FGFR1 is not a strict consequence of MAPK alterations. The fair discrimination of tumours on the tSNE based on the type of the secondary MAPK mutation (*i.e. BRAF vs. FGFR1*) even suggests that this entity could be further subdivided.

Genotype–morphotype correlations support the distinction from classical DMG, H3 K27-altered and from glial/glioneuronal tumours MAPK-altered: (i) whilst ependymal differentiation has been described in rare DMG H3-K27 [36], a mixed glioneuronal differentiation associated with CD34 positivity and eosinophilic granular bodies or a piloid differentiation are not yet described; (ii) only an exceptional subset of ganglioglioma grade 1 present *FGFR1* alteration, more characteristic of other glioneuronal tumours [24] and (iii) the existence of true malignant transformation in ganglioglioma is a matter of debate. The majority of reported cases were published before the advent of molecular biology, reclassified in a wide spectrum of CNS WHO tumour types without a distinct methylation class [28] or more interestingly were midline-located with a co-occurring *BRAF* and H3-K27M mutations [11, 21–23, 26, 29, 30, 44]. In the unified methylation class that we describe, the radiological and histopathological presentations are thus highly heterogeneous including tumours with mixed glioneuronal or pilocytic differentiation, and do not always fulfil a strict diagnostic criterion of DMG. Indeed, these tumours are less diffuse, with a frequent nodular to circumscribed radiological aspect (91% for *BRAF*^*MUT*^ and 78% for *FGFR1*^*MUT*^ DMG) and calcifications.

Several other clinical and biological characteristics support the individualization of this new entity from classical *DMG, H3 K27-altered*. First, OS is significantly different from classical DMG_K27, with a median around three years for both *FGFR1*^*MUT*^ and *BRAF*^*MUT*^ H3.3K27M DMG.

Moreover, our multivariate analysis demonstrates for the first time that the presence of these mutations is an independent prognostic factor for improved OS in DMG_K27. Previously, Picca et al*.* and Schüller et al. showed in a small populations (*n* = 6 or *n* = 7), by univariate analysis and without taking into account *TP53* status, that DMG patients with *FGFR1*^*MUT*^ have a better survival [25, 31]. The identification of a new subtype of *DMG H3 K27-altered* with longer survival is also a step forward for clinical research, highlighting the need for patient’s stratification in trials or at least molecular documentation of the cases.

Patients from Necker/Gustave Roussy cohort with *BRAF/FGFR1-mutated* DMG_K27 received, over a large period of time, quite heterogeneous treatment which did not allow specific statistical conclusion. It thus remains to be defined whether these patients could respond to a targeted therapy against BRAF^V600E^ or FGFR1.

Another meaningful difference is that DMG_K27-BRAF/FGFR1 are more frequent in the thalamus than the brainstem compared to DMG from the H3.3-K27M subtype. The age at diagnosis also differs according to the presence of the MAPK alteration. The age at onset for *FGFR1*^*MUT*^ DMG is significantly higher (median 14.8 years) and to our knowledge no adults were affected by a H3.3-K27M *BRAF*-mutated gliomas. In this new subtype, some heterogeneity remains present at various level between DMG H3K27M *BRAF* or *FGFR1-mutated*. This finding cannot be presently explained, but it may also point towards different oncogenesis, which could be individualised in the future studies.

We also investigated gene expression in these tumours and observed a senescence signature including an up-regulation of *CDKN1A* (P21) specific to DMG_K27-MAPK which is usually more present in paediatric LGGs. LGGs are characterised by an over-activated MAPK signalling in consequence to oncogenic alteration of *BRAF* or *FGFR1* [15] and the main hypothesis for a slow tumour evolution in LGG is based on the induction of oncogene-induced senescence which gives a growth advantage in a restricted window during brain development [7, 8, 27, 35, 40]. Senescence triggered via the P53/P21 axis could in part explain the slower tumour evolution in DMG_K27-MAPK. Of note, the DMG_K27-BRAF/FGFR1 share other characteristics with LGGs like calcifications and preferential association of mutations: *FGFR1* with *NF1*, *PI3KCA*, *PTPN11* [9, 17, 32, 38]. We also demonstrated in one case from this new DMG subtype harbouring *BRAF*^*V600E*^, that H3.3K27M was the first mutational event in its oncogenesis. Thus, *BRAF* and *FGFR1* mutations would be secondary driver events in the oncogenesis of these tumours and could give a proliferative advantage to the H3.3-K27M ancestral clone in a specific developmental window similarly to paediatric LGGs. Extending the analysis of sequential acquisition of mutations in DMG_K27-MAPK oncogenesis will be essential for designing future therapeutic interventions.

In conclusion, we have identified a fifth subtype of *DMG, H3 K27-altered* that we named ‘*DMG H3 K27 and BRAF/FGFR1 co-altered*’ (DMG_K27- BRAF/FGFR1), which harbours specific clinical and biological characteristics. We hypothesise that the better OS of DMG_K27-BRAF/FGFR1 compared to other DMG_K27 could be the result of both a specific cell origin and the oncogene-induced senescence. Individualization of this subtype is of importance for the interpretation of trials and affected patients may deserve specific treatment strategies.

### Supplementary Information

Below is the link to the electronic supplementary material.

Supplementary file1 (PPTX 4804 KB)

Supplementary file2 (XLSX 20 KB)

## Data Availability

DNA methylation data analysed in the current study are accessible in the ArrayExpress database at EMBL-EBI under accession number E-MTAB-13471 [https://www.ebi.ac.uk/array express/]. RNAseq data were deposited in the European Genome-Phenome Archive (EGA) [https://ega-archive.org/].

## References

[1] Bender S, Tang Y, Lindroth AM, Hovestadt V, Jones DTW, Kool M, Zapatka M, Northcott PA, Sturm D, Wang W, Radlwimmer B, Højfeldt JW, Truffaux N, Castel D, Schubert S, Ryzhova M, Şeker-Cin H, Gronych J, Johann PD, Stark S, Meyer J, Milde T, Schuhmann M, Ebinger M, Monoranu C-M, Ponnuswami A, Chen S, Jones C, Witt O, Collins VP, von Deimling A, Jabado N, Puget S, Grill J, Helin K, Korshunov A, Lichter P, Monje M, Plass C, Cho Y-J, Pfister SM (2013). Reduced H3K27me3 and DNA Hypomethylation Are Major Drivers of Gene Expression in K27M Mutant Pediatric High-Grade Gliomas. Cancer Cell.

[2] Capper D, Jones DTW, Sill M, Hovestadt V, Schrimpf D, Sturm D, Koelsche C, Sahm F, Chavez L, Reuss DE, Kratz A, Wefers AK, Huang K, Pajtler KW, Schweizer L, Stichel D, Olar A, Engel NW, Lindenberg K, Harter PN, Braczynski AK, Plate KH, Dohmen H, Garvalov BK, Coras R, Hölsken A, Hewer E, Bewerunge-Hudler M, Schick M, Fischer R, Beschorner R, Schittenhelm J, Staszewski O, Wani K, Varlet P, Pages M, Temming P, Lohmann D, Selt F, Witt H, Milde T, Witt O, Aronica E, Giangaspero F, Rushing E, Scheurlen W, Geisenberger C, Rodriguez FJ, Becker A, Preusser M, Haberler C, Bjerkvig R, Cryan J, Farrell M, Deckert M, Hench J, Frank S, Serrano J, Kannan K, Tsirigos A, Brück W, Hofer S, Brehmer S, Seiz-Rosenhagen M, Hänggi D, Hans V, Rozsnoki S, Hansford JR, Kohlhof P, Kristensen BW, Lechner M, Lopes B, Mawrin C, Ketter R, Kulozik A, Khatib Z, Heppner F, Koch A, Jouvet A, Keohane C, Mühleisen H, Mueller W, Pohl U, Prinz M, Benner A, Zapatka M, Gottardo NG, Driever PH, Kramm CM, Müller HL, Rutkowski S, von Hoff K, Frühwald MC, Gnekow A, Fleischhack G, Tippelt S, Calaminus G, Monoranu C-M, Perry A, Jones C, Jacques TS, Radlwimmer B, Gessi M, Pietsch T, Schramm J, Schackert G, Westphal M, Reifenberger G, Wesseling P, Weller M, Collins VP, Blümcke I, Bendszus M, Debus J, Huang A, Jabado N, Northcott PA, Paulus W, Gajjar A, Robinson GW, Taylor MD, Jaunmuktane Z, Ryzhova M, Platten M, Unterberg A, Wick W, Karajannis MA, Mittelbronn M, Acker T, Hartmann C, Aldape K, Schüller U, Buslei R, Lichter P, Kool M, Herold-Mende C, Ellison DW, Hasselblatt M, Snuderl M, Brandner S, Korshunov A, von Deimling A, Pfister SM (2018). DNA methylation-based classification of central nervous system tumours. Nature.

[3] Castel D, Kergrohen T, Tauziède-Espariat A, Mackay A, Ghermaoui S, Lechapt E, Pfister SM, Kramm CM, Boddaert N, Blauwblomme T, Puget S, Beccaria K, Jones C, Jones DTW, Varlet P, Grill J, Debily M-A (2020). Histone H3 wild-type DIPG/DMG overexpressing EZHIP extend the spectrum diffuse midline gliomas with PRC2 inhibition beyond H3–K27M mutation. Acta Neuropathol (Berl).

[4] Castel D, Philippe C, Calmon R, Le Dret L, Truffaux N, Boddaert N, Pagès M, Taylor KR, Saulnier P, Lacroix L, Mackay A, Jones C, Sainte-Rose C, Blauwblomme T, Andreiuolo F, Puget S, Grill J, Varlet P, Debily M-A (2015). Histone H3F3A and HIST1H3B K27M mutations define two subgroups of diffuse intrinsic pontine gliomas with different prognosis and phenotypes. Acta Neuropathol (Berl).

[5] Chan K-M, Fang D, Gan H, Hashizume R, Yu C, Schroeder M, Gupta N, Mueller S, James CD, Jenkins R, Sarkaria J, Zhang Z (2013). The histone H3.3K27M mutation in pediatric glioma reprograms H3K27 methylation and gene expression. Genes Dev.

[6] Childs BG, Baker DJ, Kirkland JL, Campisi J, Deursen JM (2014). Senescence and apoptosis: dueling or complementary cell fates?. EMBO Rep.

[7] Gnekow AK, Walker DA, Kandels D, Picton S, Perilongo G, Grill J, Stokland T, Sandstrom PE, Warmuth-Metz M, Pietsch T, Giangaspero F, Schmidt R, Faldum A, Kilmartin D, De Paoli A, De Salvo GL, Gnekow AK, Slavc I, Perilongo G, Picton S, Walker D, Stokland T, Sandstrom PE, Clausen N, Arola M, Jonsson OG, Cruz O, Navajas A, Teijeiro A, Grill J, Kalifa C, Raquin M-A, Verlooy J, Hans V, Pietsch T, Scheurlen W, Hainfellner J, Giangaspero F, Ironside J, Robson K, Skullerud K, Scheie D, Nn R-M, Jouvet A, Figarella-Branger D, Lellouch-Toubiana A, Warmuth-Metz M, Prayer D, Calderone M, Jaspan T, Bakke SJ, Vazquez E, Couanet D, Kortmann RD, Diekmann K, Scarzello G, Taylor R, Lote K, Giralt J, Carrie C, Habrand JL, Soerensen N, Czech T, Chumas P, Gustavson B, Zerah M, Wabbels B, Pinello ML, Fielder A, Simmons I, Christoffersen T, Calaminus G, Brockmann K, Straeter R, Ebinger F, Hernaiz-Driever P, Lackner H, Kennedy C, Glaser A, Stromberg B, Indiano JM, Rodary C, Bouffet E, Frappaz D, Faldum A, Emser A, De Salvo GL, Stephens S, Machin D, Le Deley M-C, Egeland T, Freemann C, Schrappe M, Sposto R (2017). A European randomised controlled trial of the addition of etoposide to standard vincristine and carboplatin induction as part of an 18-month treatment programme for childhood (≤16 years) low grade glioma – A final report. Eur J Cancer.

[8] Jacob K, Quang-Khuong D-A, Jones DTW, Witt H, Lambert S, Albrecht S, Witt O, Vezina C, Shirinian M, Faury D, Garami M, Hauser P, Klekner A, Bognar L, Farmer J-P, Montes J-L, Atkinson J, Hawkins C, Korshunov A, Collins VP, Pfister SM, Tabori U, Jabado N (2011). Genetic Aberrations Leading to MAPK Pathway Activation Mediate Oncogene-Induced Senescence in Sporadic Pilocytic Astrocytomas. Clin Cancer Res.

[9] Johnson DR, Giannini C, Jenkins RB, Kim DK, Kaufmann TJ (2019). Plenty of calcification: imaging characterization of polymorphous low-grade neuroepithelial tumor of the young. Neuroradiology.

[10] Kergrohen T, Castel D, Le Teuff G, Tauziède-Espariat A, Lechapt-Zalcman E, Nysom K, Blomgren K, Leblond P, Bertozzi A-I, De Carli E, Faure-Conter C, Chappe C, Entz-Werlé N, Moussa A, Ghermaoui S, Barret E, Picot S, Sabourin-Cousin M, Beccaria K, Vassal G, Varlet P, Puget S, Grill J, Debily M-A (2021) Copy-number alterations reshape the classification of diffuse intrinsic pontine gliomas. First exome sequencing results of the BIOMEDE trial. medRxiv. 10.1101/2021.04.29.21256183

[11] Kleinschmidt-DeMasters BK, Donson A, Foreman NK, Dorris K (2017). H3 K27M Mutation in Gangliogliomas can be Associated with Poor Prognosis. Brain Pathol Zurich Switz.

[12] Lew ED, Furdui CM, Anderson KS, Schlessinger J (2009). The Precise Sequence of FGF Receptor Autophosphorylation Is Kinetically Driven and Is Disrupted by Oncogenic Mutations. Sci Signal.

[13] Lewis PW, Muller MM, Koletsky MS, Cordero F, Lin S, Banaszynski LA, Garcia BA, Muir TW, Becher OJ, Allis CD (2013). Inhibition of PRC2 Activity by a Gain-of-Function H3 Mutation Found in Pediatric Glioblastoma. Science.

[14] Liu X, Ding J, Meng L (2018). Oncogene-induced senescence: a double edged sword in cancer. Acta Pharmacol Sin.

[15] Louis DN, Perry A, Reifenberger G, von Deimling A, Figarella-Branger D, Cavenee WK, Ohgaki H, Wiestler OD, Kleihues P, Ellison DW (2016). The 2016 World Health Organization Classification of Tumors of the Central Nervous System: a summary. Acta Neuropathol (Berl).

[16] Louis DN, Perry A, Wesseling P, Brat DJ, Cree IA, Figarella-Branger D, Hawkins C, Ng HK, Pfister SM, Reifenberger G, Soffietti R, von Deimling A, Ellison DW (2021). The 2021 WHO Classification of Tumors of the Central Nervous System: a summary. Neuro-Oncol.

[17] Lucas C-HG, Gupta R, Doo P, Lee JC, Cadwell CR, Ramani B, Hofmann JW, Sloan EA, Kleinschmidt-DeMasters BK, Lee HS, Wood MD, Grafe M, Born D, Vogel H, Salamat S, Puccetti D, Scharnhorst D, Samuel D, Cooney T, Cham E, Jin L, Khatib Z, Maher O, Chamyan G, Brathwaite C, Bannykh S, Mueller S, Kline CN, Banerjee A, Reddy A, Taylor JW, Clarke JL, Oberheim Bush NA, Butowski N, Gupta N, Auguste KI, Sun PP, Roland JL, Raffel C, Aghi MK, Theodosopoulos P, Chang E, Hervey-Jumper S, Phillips JJ, Pekmezci M, Bollen AW, Tihan T, Chang S, Berger MS, Perry A, Solomon DA (2020). Comprehensive analysis of diverse low-grade neuroepithelial tumors with FGFR1 alterations reveals a distinct molecular signature of rosette-forming glioneuronal tumor. Acta Neuropathol Commun.

[18] Mackay A, Burford A, Carvalho D, Izquierdo E, Fazal-Salom J, Taylor KR, Bjerke L, Clarke M, Vinci M, Nandhabalan M, Temelso S, Popov S, Molinari V, Raman P, Waanders AJ, Han HJ, Gupta S, Marshall L, Zacharoulis S, Vaidya S, Mandeville HC, Bridges LR, Martin AJ, Al-Sarraj S, Chandler C, Ng H-K, Li X, Mu K, Trabelsi S, Brahim DH-B, Kisljakov AN, Konovalov DM, Moore AS, Carcaboso AM, Sunol M, de Torres C, Cruz O, Mora J, Shats LI, Stavale JN, Bidinotto LT, Reis RM, Entz-Werle N, Farrell M, Cryan J, Crimmins D, Caird J, Pears J, Monje M, Debily M-A, Castel D, Grill J, Hawkins C, Nikbakht H, Jabado N, Baker SJ, Pfister SM, Jones DTW, Fouladi M, von Bueren AO, Baudis M, Resnick A, Jones C (2017). Integrated Molecular Meta-Analysis of 1,000 Pediatric High-Grade and Diffuse Intrinsic Pontine Glioma. Cancer Cell.

[19] Mondal G, Lee JC, Ravindranathan A, Villanueva-Meyer JE, Tran QT, Allen SJ, Barreto J, Gupta R, Doo P, Van Ziffle J, Onodera C, Devine P, Grenert JP, Samuel D, Li R, Metrock LK, Jin L-W, Antony R, Alashari M, Cheshier S, Whipple NS, Bruggers C, Raffel C, Gupta N, Kline CN, Reddy A, Banerjee A, Hall MD, Mehta MP, Khatib Z, Maher OM, Brathwaite C, Pekmezci M, Phillips JJ, Bollen AW, Tihan T, Lucas JT, Broniscer A, Berger MS, Perry A, Orr BA, Solomon DA (2020). Pediatric bithalamic gliomas have a distinct epigenetic signature and frequent EGFR exon 20 insertions resulting in potential sensitivity to targeted kinase inhibition. Acta Neuropathol (Berl).

[20] Nakamura IT, Kohsaka S, Ikegami M, Ikeuchi H, Ueno T, Li K, Beyett TS, Koyama T, Shimizu T, Yamamoto N, Takahashi F, Takahashi K, Eck MJ, Mano H (2021). Comprehensive functional evaluation of variants of fibroblast growth factor receptor genes in cancer. Npj Precis Oncol.

[21] Nguyen AT, Colin C, Nanni-Metellus I, Padovani L, Maurage C-A, Varlet P, Miquel C, Uro-Coste E, Godfraind C, Lechapt-Zalcman E, Labrousse F, Gauchotte G, Silva K, Jouvet A, Figarella-Branger D, the French GENOP Network,  (2015). Evidence for *BRAF* V600E and *H3F3A* K27M double mutations in paediatric glial and glioneuronal tumours. Neuropathol Appl Neurobiol.

[22] Orillac C, Thomas C, Dastagirzada Y, Hidalgo ET, Golfinos JG, Zagzag D, Wisoff JH, Karajannis MA, Snuderl M (2016). Pilocytic astrocytoma and glioneuronal tumor with histone H3 K27M mutation. Acta Neuropathol Commun.

[23] Pagès M, Beccaria K, Boddaert N, Saffroy R, Besnard A, Castel D, Fina F, Barets D, Barret E, Lacroix L, Bielle F, Andreiuolo F, Tauziède-Espariat A, Figarella-Branger D, Puget S, Grill J, Chrétien F, Varlet P (2018). Co-occurrence of histone H3 K27M and BRAF V600E mutations in paediatric midline grade I ganglioglioma: Histone H3 K27M and BRAF V600E mutations in GG. Brain Pathol.

[24] Pekmezci M, Villanueva-Meyer JE, Goode B, Van Ziffle J, Onodera C, Grenert JP, Bastian BC, Chamyan G, Maher OM, Khatib Z, Kleinschmidt-DeMasters BK, Samuel D, Mueller S, Banerjee A, Clarke JL, Cooney T, Torkildson J, Gupta N, Theodosopoulos P, Chang EF, Berger M, Bollen AW, Perry A, Tihan T, Solomon DA (2018). The genetic landscape of ganglioglioma. Acta Neuropathol Commun.

[25] Picca A, Berzero G, Bielle F, Touat M, Savatovsky J, Polivka M, Trisolini E, Meunier S, Schmitt Y, Idbaih A, Hoang-Xuan K, Delattre J-Y, Mokhtari K, Di Stefano AL, Sanson M (2018). FGFR1 actionable mutations, molecular specificities, and outcome of adult midline gliomas. Neurology.

[26] Pratt D, Natarajan SK, Banda A, Giannini C, Vats P, Koschmann C, Mody R, Chinnaiyan A, Venneti S (2018). Circumscribed/non-diffuse histology confers a better prognosis in H3K27M-mutant gliomas. Acta Neuropathol (Berl).

[27] Raabe EH, Lim KS, Kim JM, Meeker A, Mao X, -g., Nikkhah G, Maciaczyk J, Kahlert U, Jain D, Bar E, Cohen KJ, Eberhart CG,  (2011). BRAF Activation Induces Transformation and Then Senescence in Human Neural Stem Cells: A Pilocytic Astrocytoma Model. Clin Cancer Res.

[28] Reinhardt A, Pfister K, Schrimpf D, Stichel D, Sahm F, Reuss DE, Capper D, Wefers AK, Ebrahimi A, Sill M, Felsberg J, Reifenberger G, Becker A, Prinz M, Staszewski O, Hartmann C, Schittenhelm J, Gramatzki D, Weller M, Olar A, Rushing EJ, Bergmann M, Farrell MA, Blümcke I, Coras R, Beckervordersandforth J, Kim SH, Rogerio F, Dimova PS, Niehusmann P, Unterberg A, Platten M, Pfister SM, Wick W, Herold-Mende C, Von Deimling A (2022). Anaplastic ganglioglioma—A diagnosis comprising several distinct tumour types. Neuropathol Appl Neurobiol.

[29] Rodriguez FJ, Brosnan-Cashman JA, Allen SJ, Vizcaino MA, Giannini C, Camelo-Piragua S, Webb M, Matsushita M, Wadhwani N, Tabbarah A, Hamideh D, Jiang L, Chen L, Arvanitis LD, Alnajar HH, Barber JR, Rodríguez-Velasco A, Orr B, Heaphy CM (2019). Alternative lengthening of telomeres, ATRX loss and H3–K27M mutations in histologically defined pilocytic astrocytoma with anaplasia. Brain Pathol Zurich Switz.

[30] Ryall S, Zapotocky M, Fukuoka K, Nobre L, Guerreiro Stucklin A, Bennett J, Siddaway R, Li C, Pajovic S, Arnoldo A, Kowalski PE, Johnson M, Sheth J, Lassaletta A, Tatevossian RG, Orisme W, Qaddoumi I, Surrey LF, Li MM, Waanders AJ, Gilheeney S, Rosenblum M, Bale T, Tsang DS, Laperriere N, Kulkarni A, Ibrahim GM, Drake J, Dirks P, Taylor MD, Rutka JT, Laughlin S, Shroff M, Shago M, Hazrati L-N, D’Arcy C, Ramaswamy V, Bartels U, Huang A, Bouffet E, Karajannis MA, Santi M, Ellison DW, Tabori U, Hawkins C (2020). Integrated Molecular and Clinical Analysis of 1,000 Pediatric Low-Grade Gliomas. Cancer Cell.

[31] Schüller U, Iglauer P, Dorostkar MM, Mawrin C, Herms J, Giese A, Glatzel M, Neumann JE (2021). Mutations within FGFR1 are associated with superior outcome in a series of 83 diffuse midline gliomas with H3F3A K27M mutations. Acta Neuropathol (Berl).

[32] Sievers P, Appay R, Schrimpf D, Stichel D, Reuss DE, Wefers AK, Reinhardt A, Coras R, Ruf VC, Schmid S, de Stricker K, Boldt HB, Kristensen BW, Petersen JK, Ulhøi BP, Gardberg M, Aronica E, Hasselblatt M, Brück W, Bielle F, Mokhtari K, Lhermitte B, Wick W, Herold-Mende C, Hänggi D, Brandner S, Giangaspero F, Capper D, Rushing E, Wesseling P, Pfister SM, Figarella-Branger D, von Deimling A, Sahm F, Jones DTW (2019). Rosette-forming glioneuronal tumors share a distinct DNA methylation profile and mutations in FGFR1, with recurrent co-mutation of PIK3CA and NF1. Acta Neuropathol (Berl).

[33] Sievers P, Sill M, Schrimpf D, Stichel D, Reuss DE, Sturm D, Hench J, Frank S, Krskova L, Vicha A, Zapotocky M, Bison B, Castel D, Grill J, Debily M-A, Harter PN, Snuderl M, Kramm CM, Reifenberger G, Korshunov A, Jabado N, Wesseling P, Wick W, Solomon DA, Perry A, Jacques TS, Jones C, Witt O, Pfister SM, von Deimling A, Jones DTW, Sahm F (2021). A subset of pediatric-type thalamic gliomas share a distinct DNA methylation profile, H3K27me3 loss and frequent alteration of *EGFR*. Neuro-Oncol.

[34] Solomon DA, Wood MD, Tihan T, Bollen AW, Gupta N, Phillips JJJ, Perry A (2016). Diffuse Midline Gliomas with Histone H3–K27M Mutation: A Series of 47 Cases Assessing the Spectrum of Morphologic Variation and Associated Genetic Alterations: Diffuse midline gliomas with histone H3–K27M mutation. Brain Pathol.

[35] Tabori U, Vukovic B, Zielenska M, Hawkins C, Braude I, Rutka J, Bouffet E, Squire J, Malkin D (2006). The Role of Telomere Maintenance in the Spontaneous Growth Arrest of Pediatric Low-Grade Gliomas. Neoplasia.

[36] Tauziède-Espariat A, Métais A, Mariet C, Castel D, Grill J, Saffroy R, Hasty L, Dangouloff-Ros V, Boddaert N, Benichi S, Chrétien F, Varlet P (2023). The pontine diffuse midline glioma, EGFR -subtype with ependymal features: Yet another face of diffuse midline glioma, H3K27-altered. Brain Pathol.

[37] Taylor KR, Mackay A, Truffaux N, Butterfield YS, Morozova O, Philippe C, Castel D, Grasso CS, Vinci M, Carvalho D, Carcaboso AM, de Torres C, Cruz O, Mora J, Entz-Werle N, Ingram WJ, Monje M, Hargrave D, Bullock AN, Puget S, Yip S, Jones C, Grill J (2014). Recurrent activating ACVR1 mutations in diffuse intrinsic pontine glioma. Nat Genet.

[38] Jones DTW, Hutter B, Jäger N, Korshunov A, Kool M, Warnatz H-J, Zichner T, Lambert SR, Ryzhova M, Quang DAK, Fontebasso AM, Stütz AM, Hutter S, Zuckermann M, Sturm D, Gronych J, Lasitschka B, Schmidt S, Şeker-Cin H, Witt H, Sultan M, Ralser M, Northcott PA, Hovestadt V, Bender S, Pfaff E, Stark S, Faury D, Schwartzentruber J, Majewski J, Weber UD, Zapatka M, Raeder B, Schlesner M, Worth CL, Bartholomae CC, von Kalle C, Imbusch CD, Radomski S, Lawerenz C, van Sluis P, Koster J, Volckmann R, Versteeg R, Lehrach H, Monoranu C, Winkler B, Unterberg A, Herold-Mende C, Milde T, Kulozik AE, Ebinger M, Schuhmann MU, Cho Y-J, Pomeroy SL, von Deimling A, Witt O, Taylor MD, Wolf S, Karajannis MA, Eberhart CG, Scheurlen W, Hasselblatt M, Ligon KL, Kieran MW, Korbel JO, Yaspo M-L, Brors B, Felsberg J, Reifenberger G, Collins VP, Jabado N, Eils R, Lichter P, The International Cancer Genome Consortium PedBrain Tumor Project (2013). Recurrent somatic alterations of FGFR1 and NTRK2 in pilocytic astrocytoma. Nat Genet.

[39] Visvader JE (2011). Cells of origin in cancer. Nature.

[40] Warrington NM, Woerner BM, Daginakatte GC, Dasgupta B, Perry A, Gutmann DH, Rubin JB (2007). Spatiotemporal Differences in CXCL12 Expression and Cyclic AMP Underlie the Unique Pattern of Optic Glioma Growth in Neurofibromatosis Type 1. Cancer Res.

[41] Werbrouck C, Evangelista CCS, Lobón-Iglesias M-J, Barret E, Le Teuff G, Merlevede J, Brusini R, Kergrohen T, Mondini M, Bolle S, Varlet P, Beccaria K, Boddaert N, Puget S, Grill J, Debily M-A, Castel D (2019). TP53 Pathway Alterations Drive Radioresistance in Diffuse Intrinsic Pontine Gliomas (DIPG). Clin Cancer Res.

[42] Wu G, Broniscer A, McEachron TA, Lu C, Paugh BS, Becksfort J, Qu C, Ding L, Huether R, Parker M, Zhang J, Gajjar A, Dyer MA, Mullighan CG, Gilbertson RJ, Mardis ER, Wilson RK, Downing JR, Ellison DW, Zhang J, Baker SJ (2012). Somatic Histone H3 Alterations in Paediatric Diffuse Intrinsic Pontine Gliomas and Non-Brainstem Glioblastomas. Nat Genet.

[43] Yoon K (2004). Fibroblast Growth Factor Receptor Signaling Promotes Radial Glial Identity and Interacts with Notch1 Signaling in Telencephalic Progenitors. J Neurosci.

[44] Zanello M, Pages M, Tauziède-Espariat A, Saffroy R, Puget S, Lacroix L, Dezamis E, Devaux B, Chrétien F, Andreiuolo F, Sainte-Rose C, Zerah M, Dhermain F, Dumont S, Louvel G, Meder J-F, Grill J, Dufour C, Pallud J, Varlet P (2016). Clinical, Imaging, Histopathological and Molecular Characterization of Anaplastic Ganglioglioma. J Neuropathol Exp Neurol.

[45] Zheng L, Gong J, Yu T, Zou Y, Zhang M, Nie L, Chen X, Yue Q, Liu Y, Mao Q, Zhou Q, Chen N (2022). Diffuse Midline Gliomas With Histone H3 K27M Mutation in Adults and Children: A Retrospective Series of 164 Cases. Am J Surg Pathol.

