# Supplementary material for: A new subtype of diffuse midline glioma, H3 K27 and BRAF/FGFR1 co-altered: a clinico-radiological and histomolecular characterisation
Source: Acta Neuropathol. 2023 Dec 8;147(1):2. doi: 10.1007/s00401-023-02651-4 (PMC10709479; doi:10.1007/s00401-023-02651-4)
Supplement: Supplementary file 1 — Supplementary file1 (PPTX 4804 KB) [file 401_2023_2651_MOESM1_ESM.pptx]

## Slide 1
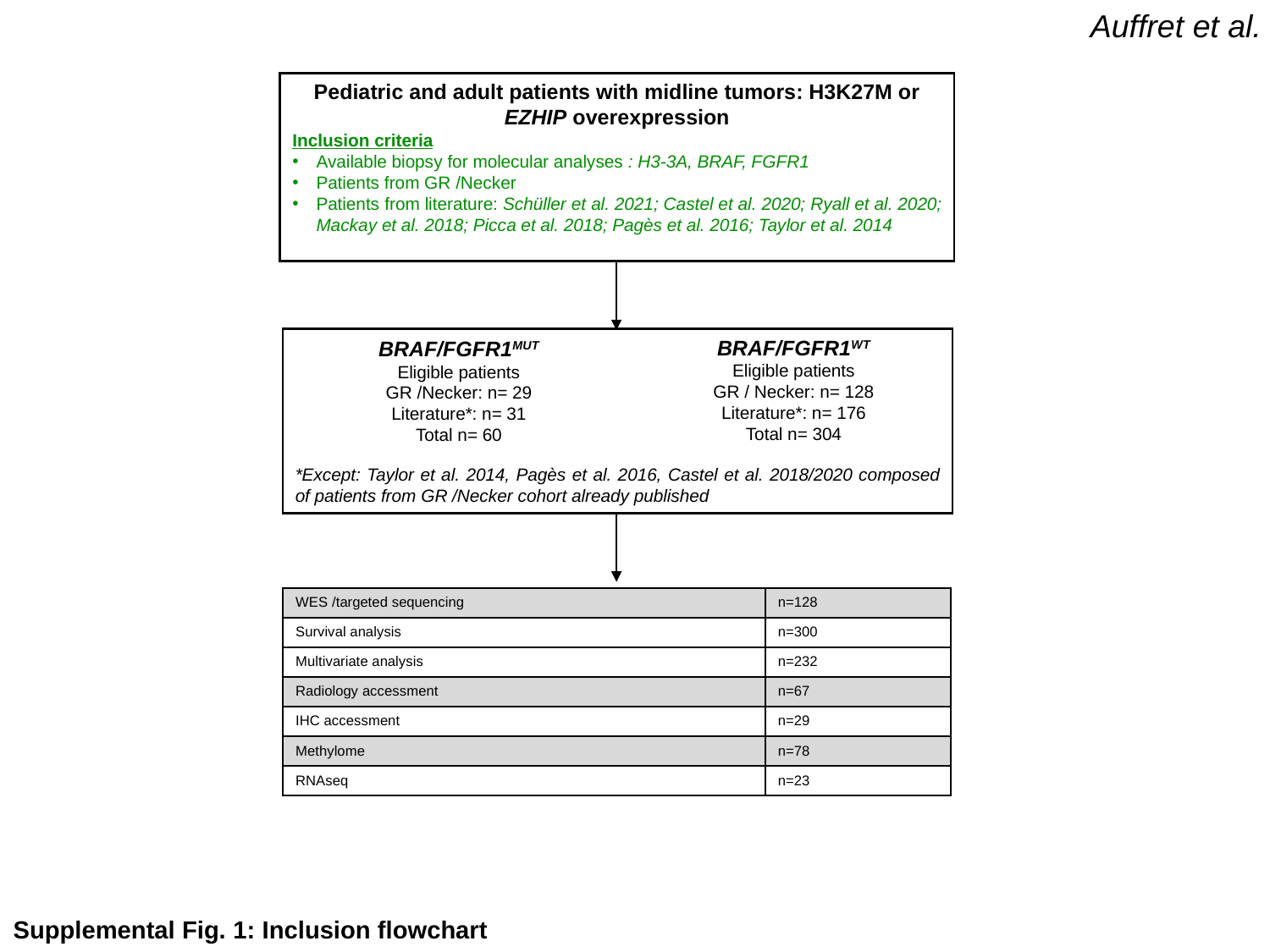

Auffret et al.
Pediatric and adult patients with midline tumors: H3K27M or EZHIP overexpression
Inclusion criteria
Available biopsy for molecular analyses : H3-3A, BRAF, FGFR1
Patients from GR /Necker
Patients from literature: Schüller et al. 2021; Castel et al. 2020; Ryall et al. 2020; Mackay et al. 2018; Picca et al. 2018; Pagès et al. 2016; Taylor et al. 2014
BRAF/FGFR1WT
Eligible patients
GR / Necker: n= 128
Literature*: n= 176
Total n= 304
BRAF/FGFR1MUT
Eligible patients
GR /Necker: n= 29
Literature*: n= 31
Total n= 60
*Except: Taylor et al. 2014, Pagès et al. 2016, Castel et al. 2018/2020 composed of patients from GR /Necker cohort already published
| WES /targeted sequencing | n=128 |
| --- | --- |
| Survival analysis | n=300 |
| Multivariate analysis | n=232 |
| Radiology accessment | n=67 |
| IHC accessment | n=29 |
| Methylome | n=78 |
| RNAseq | n=23 |
Supplemental Fig. 1: Inclusion flowchart

## Slide 2
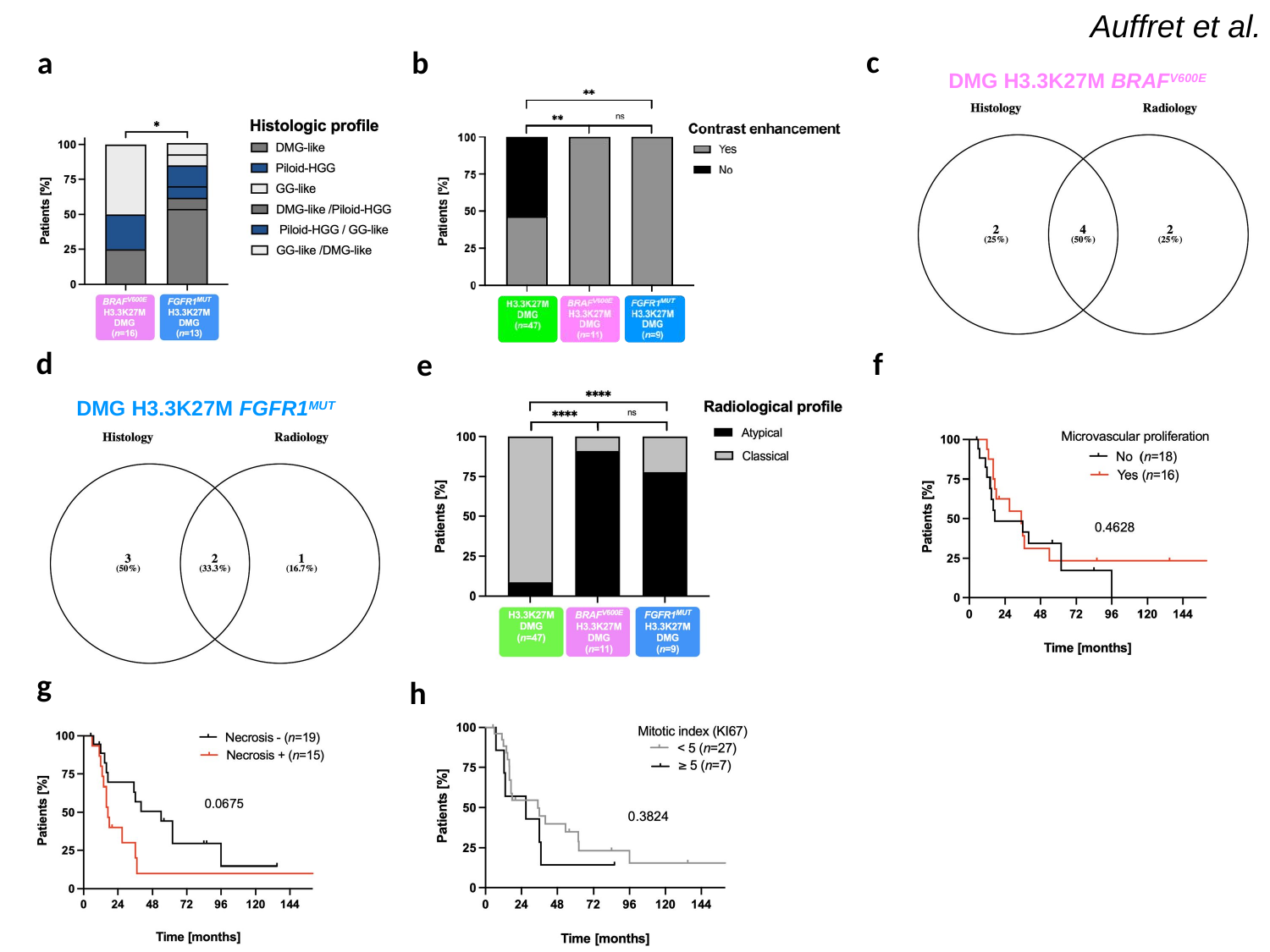

Auffret et al.
c
a
b
DMG H3.3K27M BRAFV600E
d
f
e
DMG H3.3K27M FGFR1MUT
g
h

## Slide 3
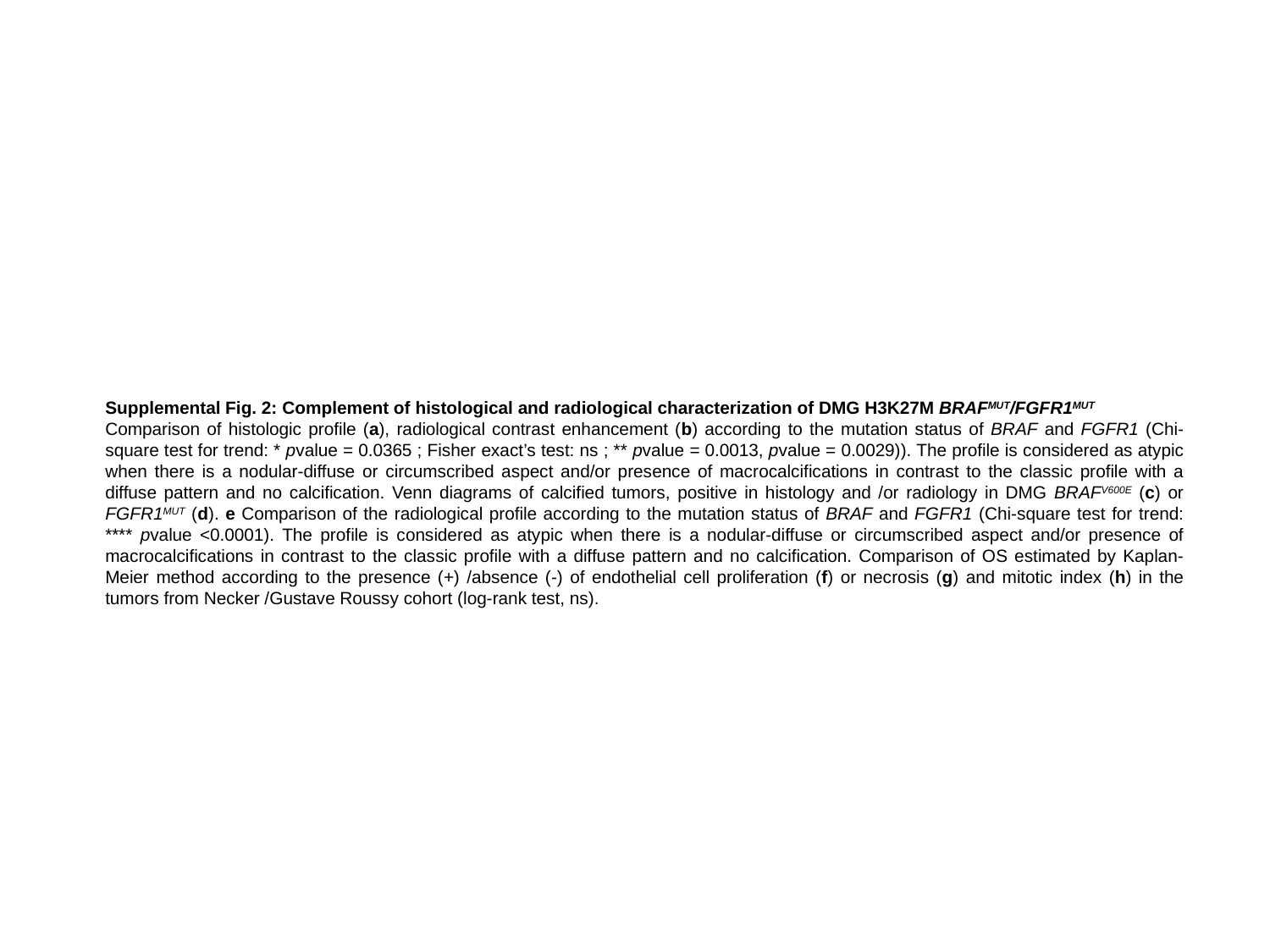

Supplemental Fig. 2: Complement of histological and radiological characterization of DMG H3K27M BRAFMUT/FGFR1MUT
Comparison of histologic profile (a), radiological contrast enhancement (b) according to the mutation status of BRAF and FGFR1 (Chi-square test for trend: * pvalue = 0.0365 ; Fisher exact’s test: ns ; ** pvalue = 0.0013, pvalue = 0.0029)). The profile is considered as atypic when there is a nodular-diffuse or circumscribed aspect and/or presence of macrocalcifications in contrast to the classic profile with a diffuse pattern and no calcification. Venn diagrams of calcified tumors, positive in histology and /or radiology in DMG BRAFV600E (c) or FGFR1MUT (d). e Comparison of the radiological profile according to the mutation status of BRAF and FGFR1 (Chi-square test for trend: **** pvalue <0.0001). The profile is considered as atypic when there is a nodular-diffuse or circumscribed aspect and/or presence of macrocalcifications in contrast to the classic profile with a diffuse pattern and no calcification. Comparison of OS estimated by Kaplan-Meier method according to the presence (+) /absence (-) of endothelial cell proliferation (f) or necrosis (g) and mitotic index (h) in the tumors from Necker /Gustave Roussy cohort (log-rank test, ns).

## Slide 4
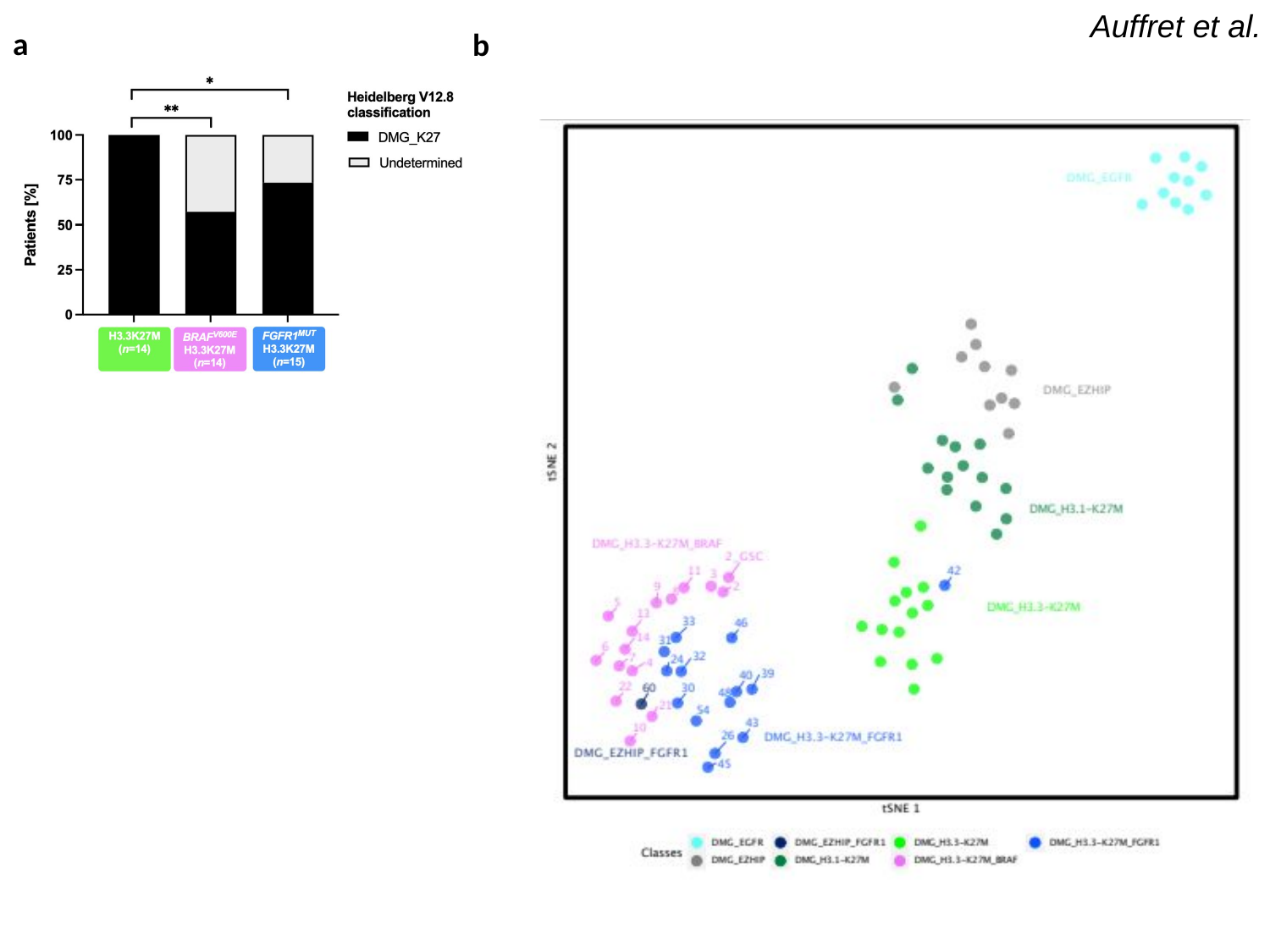

Auffret et al.
a
b

## Slide 5
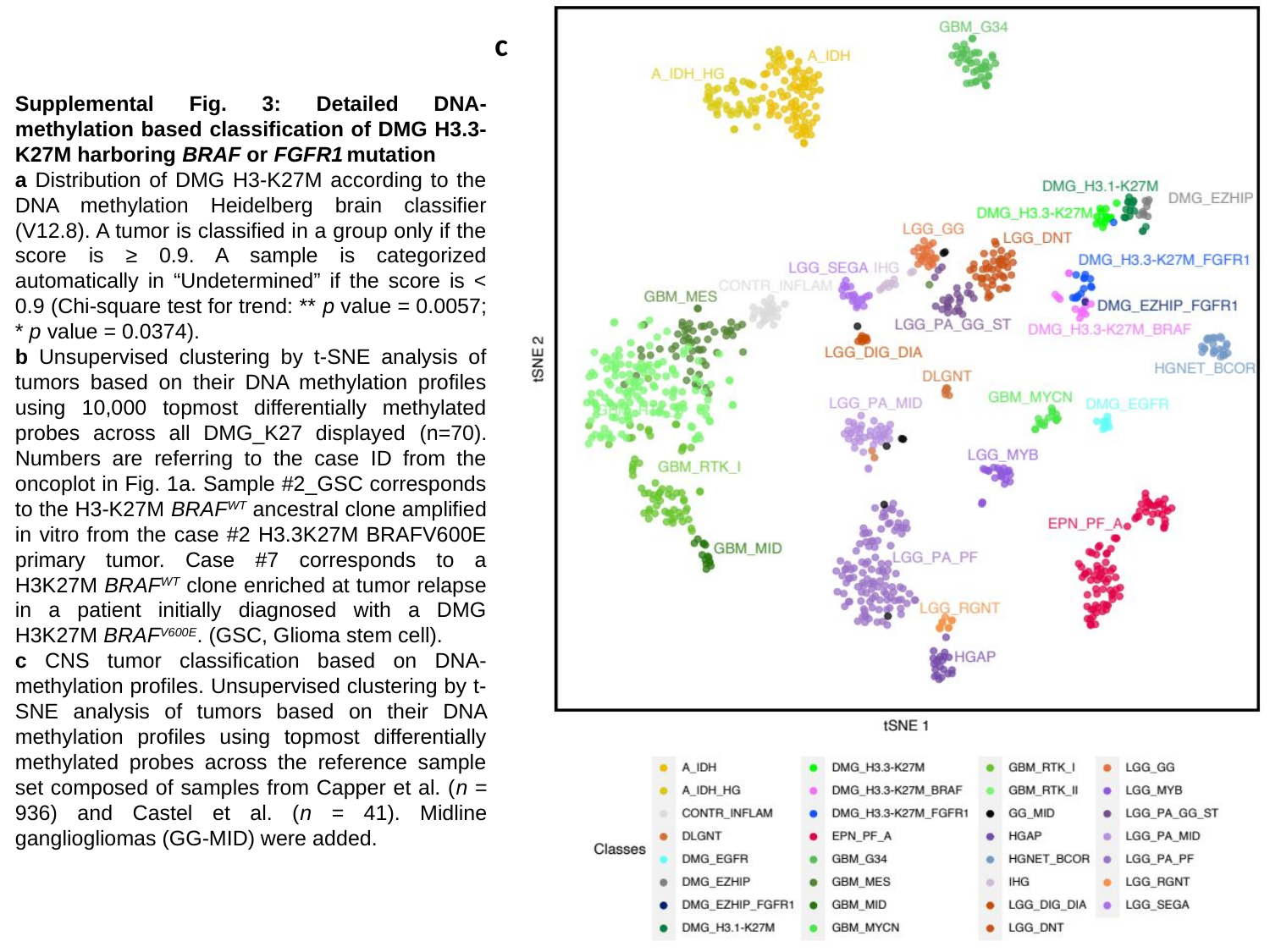

c
Supplemental Fig. 3: Detailed DNA-methylation based classification of DMG H3.3-K27M harboring BRAF or FGFR1 mutation
a Distribution of DMG H3-K27M according to the DNA methylation Heidelberg brain classifier (V12.8). A tumor is classified in a group only if the score is ≥ 0.9. A sample is categorized automatically in “Undetermined” if the score is < 0.9 (Chi-square test for trend: ** p value = 0.0057; * p value = 0.0374).
b Unsupervised clustering by t-SNE analysis of tumors based on their DNA methylation profiles using 10,000 topmost differentially methylated probes across all DMG_K27 displayed (n=70). Numbers are referring to the case ID from the oncoplot in Fig. 1a. Sample #2_GSC corresponds to the H3-K27M BRAFWT ancestral clone amplified in vitro from the case #2 H3.3K27M BRAFV600E primary tumor. Case #7 corresponds to a H3K27M BRAFWT clone enriched at tumor relapse in a patient initially diagnosed with a DMG H3K27M BRAFV600E. (GSC, Glioma stem cell).
c CNS tumor classification based on DNA-methylation profiles. Unsupervised clustering by t-SNE analysis of tumors based on their DNA methylation profiles using topmost differentially methylated probes across the reference sample set composed of samples from Capper et al. (n = 936) and Castel et al. (n = 41). Midline gangliogliomas (GG-MID) were added.

## Slide 6
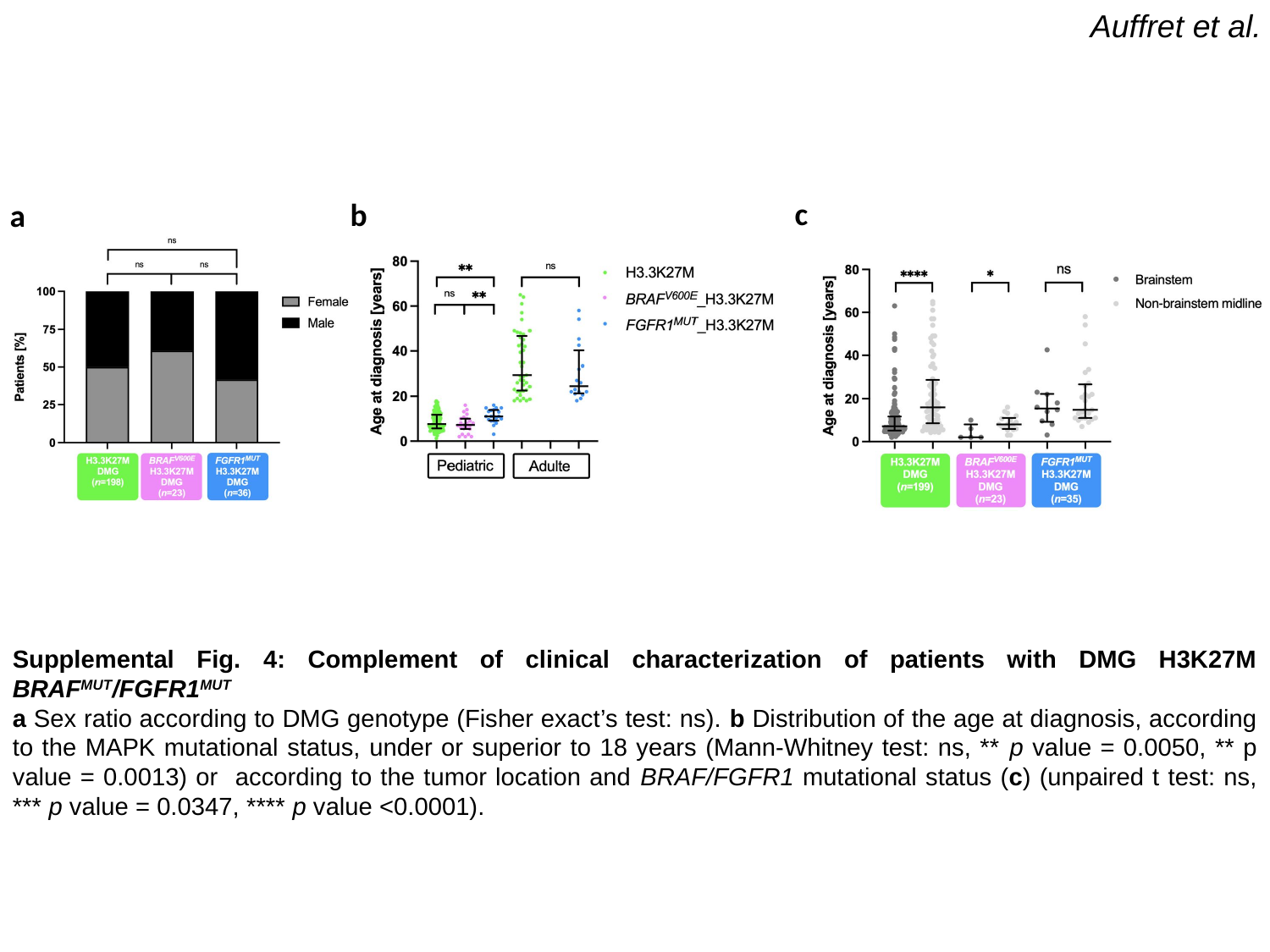

Auffret et al.
c
b
a
Supplemental Fig. 4: Complement of clinical characterization of patients with DMG H3K27M BRAFMUT/FGFR1MUT
a Sex ratio according to DMG genotype (Fisher exact’s test: ns). b Distribution of the age at diagnosis, according to the MAPK mutational status, under or superior to 18 years (Mann-Whitney test: ns, ** p value = 0.0050, ** p value = 0.0013) or according to the tumor location and BRAF/FGFR1 mutational status (c) (unpaired t test: ns, *** p value = 0.0347, **** p value <0.0001).

## Slide 7
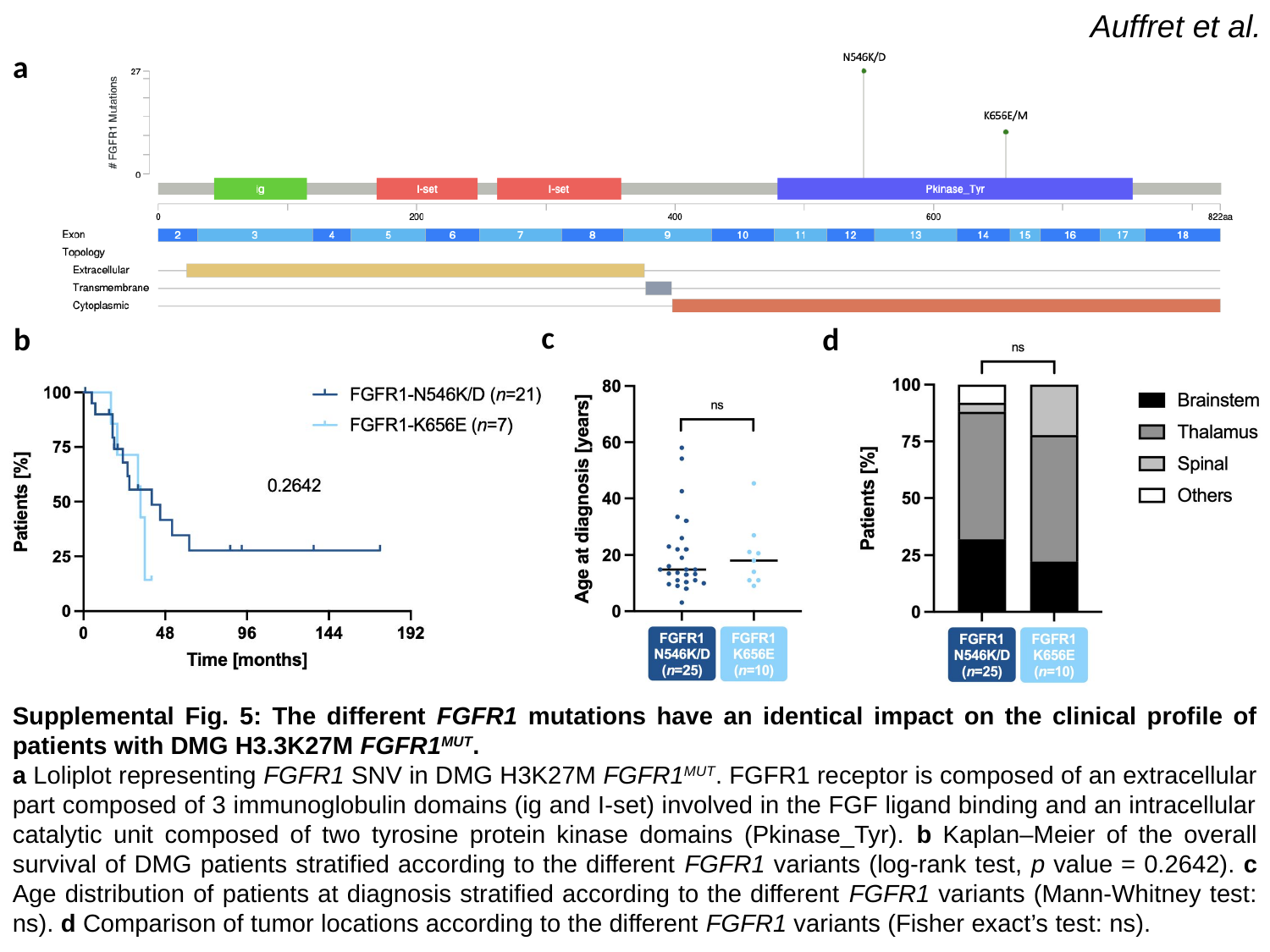

Auffret et al.
a
c
d
b
Supplemental Fig. 5: The different FGFR1 mutations have an identical impact on the clinical profile of patients with DMG H3.3K27M FGFR1MUT.
a Loliplot representing FGFR1 SNV in DMG H3K27M FGFR1MUT. FGFR1 receptor is composed of an extracellular part composed of 3 immunoglobulin domains (ig and I-set) involved in the FGF ligand binding and an intracellular catalytic unit composed of two tyrosine protein kinase domains (Pkinase_Tyr). b Kaplan–Meier of the overall survival of DMG patients stratified according to the different FGFR1 variants (log-rank test, p value = 0.2642). c Age distribution of patients at diagnosis stratified according to the different FGFR1 variants (Mann-Whitney test: ns). d Comparison of tumor locations according to the different FGFR1 variants (Fisher exact’s test: ns).

## Slide 8
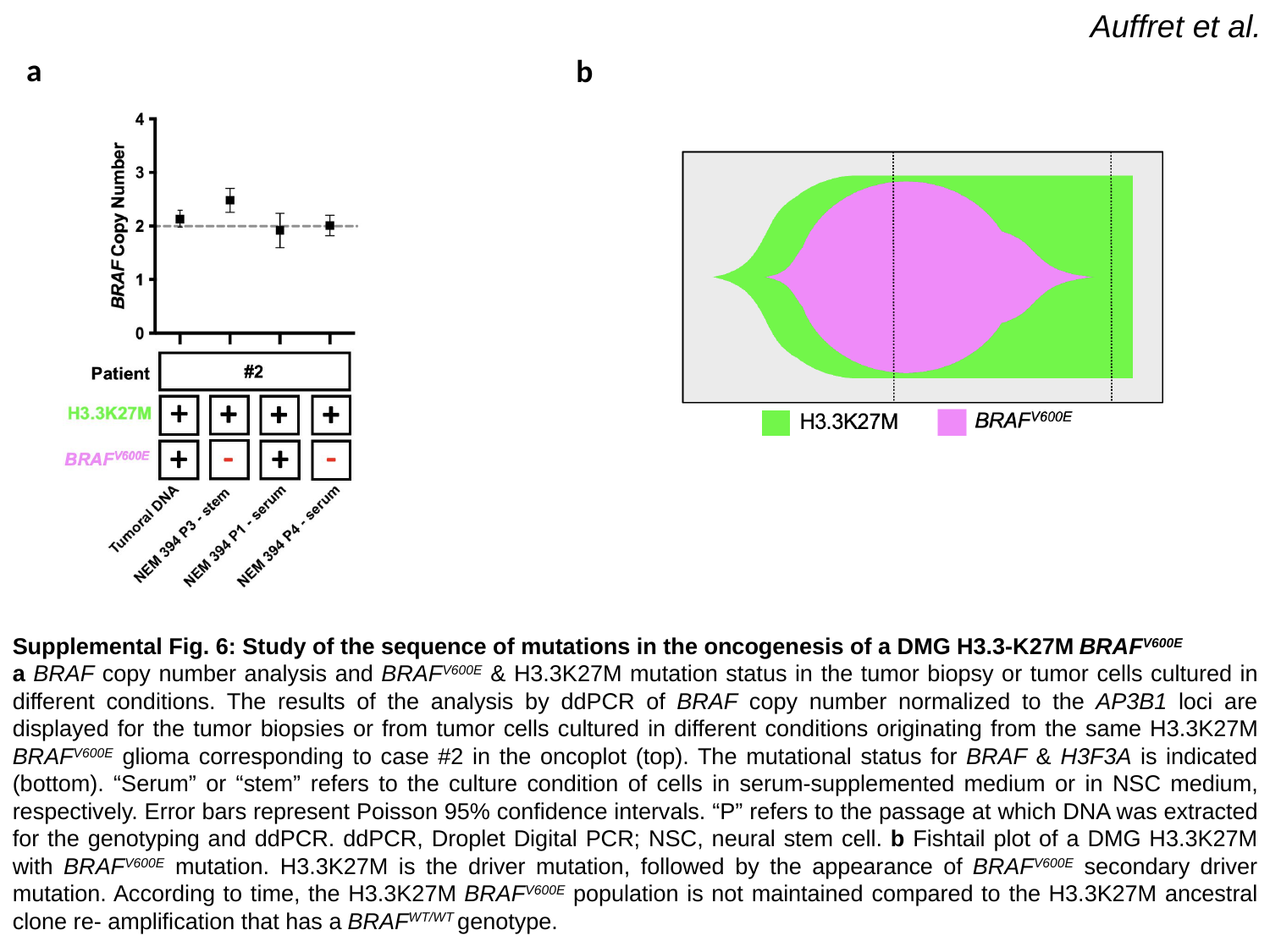

Auffret et al.
a
b
Supplemental Fig. 6: Study of the sequence of mutations in the oncogenesis of a DMG H3.3-K27M BRAFV600E
a BRAF copy number analysis and BRAFV600E & H3.3K27M mutation status in the tumor biopsy or tumor cells cultured in different conditions. The results of the analysis by ddPCR of BRAF copy number normalized to the AP3B1 loci are displayed for the tumor biopsies or from tumor cells cultured in different conditions originating from the same H3.3K27M BRAFV600E glioma corresponding to case #2 in the oncoplot (top). The mutational status for BRAF & H3F3A is indicated (bottom). “Serum” or “stem” refers to the culture condition of cells in serum-supplemented medium or in NSC medium, respectively. Error bars represent Poisson 95% confidence intervals. “P” refers to the passage at which DNA was extracted for the genotyping and ddPCR. ddPCR, Droplet Digital PCR; NSC, neural stem cell. b Fishtail plot of a DMG H3.3K27M with BRAFV600E mutation. H3.3K27M is the driver mutation, followed by the appearance of BRAFV600E secondary driver mutation. According to time, the H3.3K27M BRAFV600E population is not maintained compared to the H3.3K27M ancestral clone re- amplification that has a BRAFWT/WT genotype.

## Slide 9
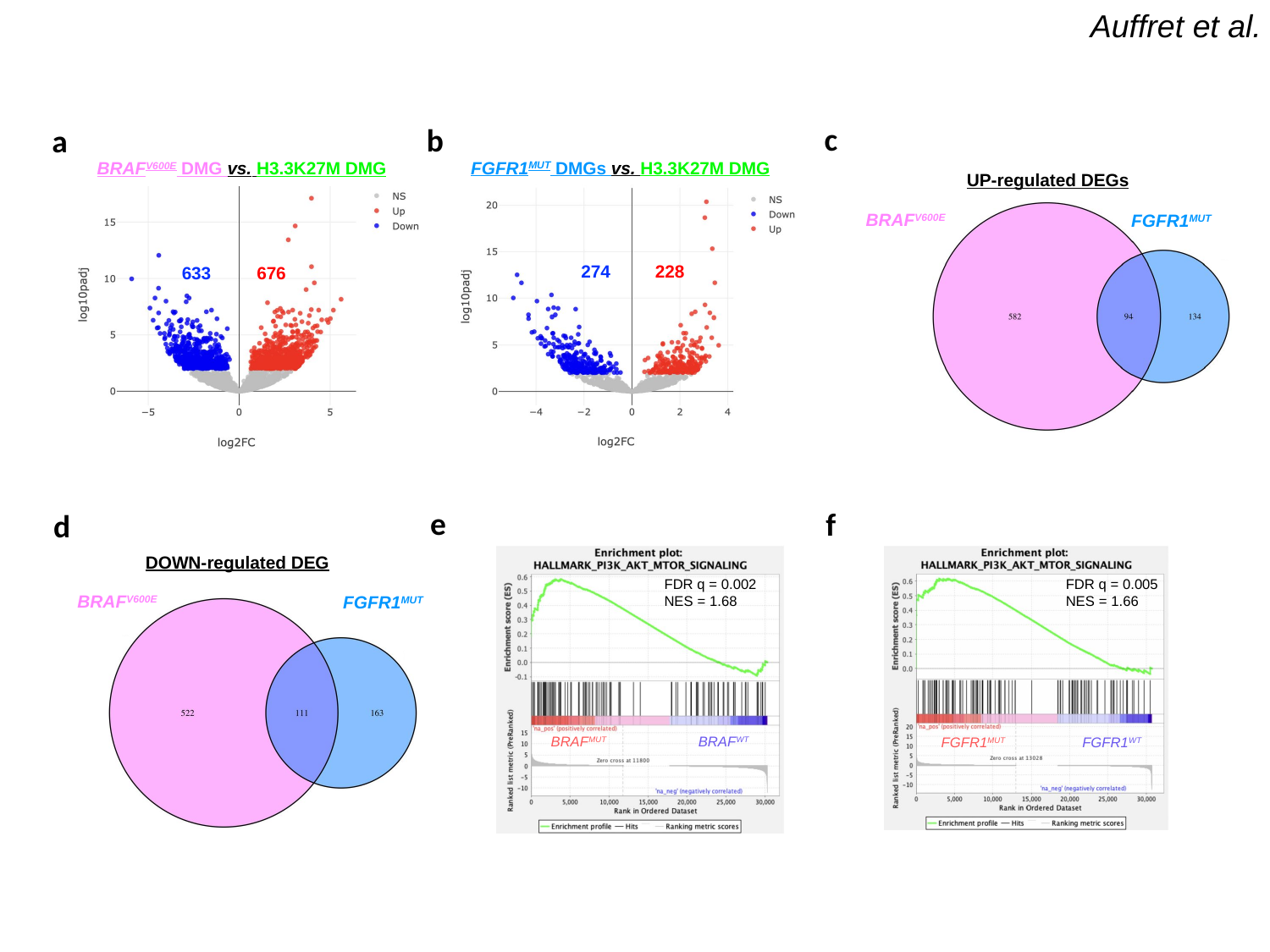

Auffret et al.
c
b
a
FGFR1MUT DMGs vs. H3.3K27M DMG
BRAFV600E DMG vs. H3.3K27M DMG
UP-regulated DEGs
BRAFV600E
FGFR1MUT
274
228
633
676
e
f
d
DOWN-regulated DEG
FDR q = 0.005
NES = 1.66
FDR q = 0.002
NES = 1.68
BRAFV600E
FGFR1MUT
BRAFMUT
BRAFWT
FGFR1MUT
FGFR1WT

## Slide 10
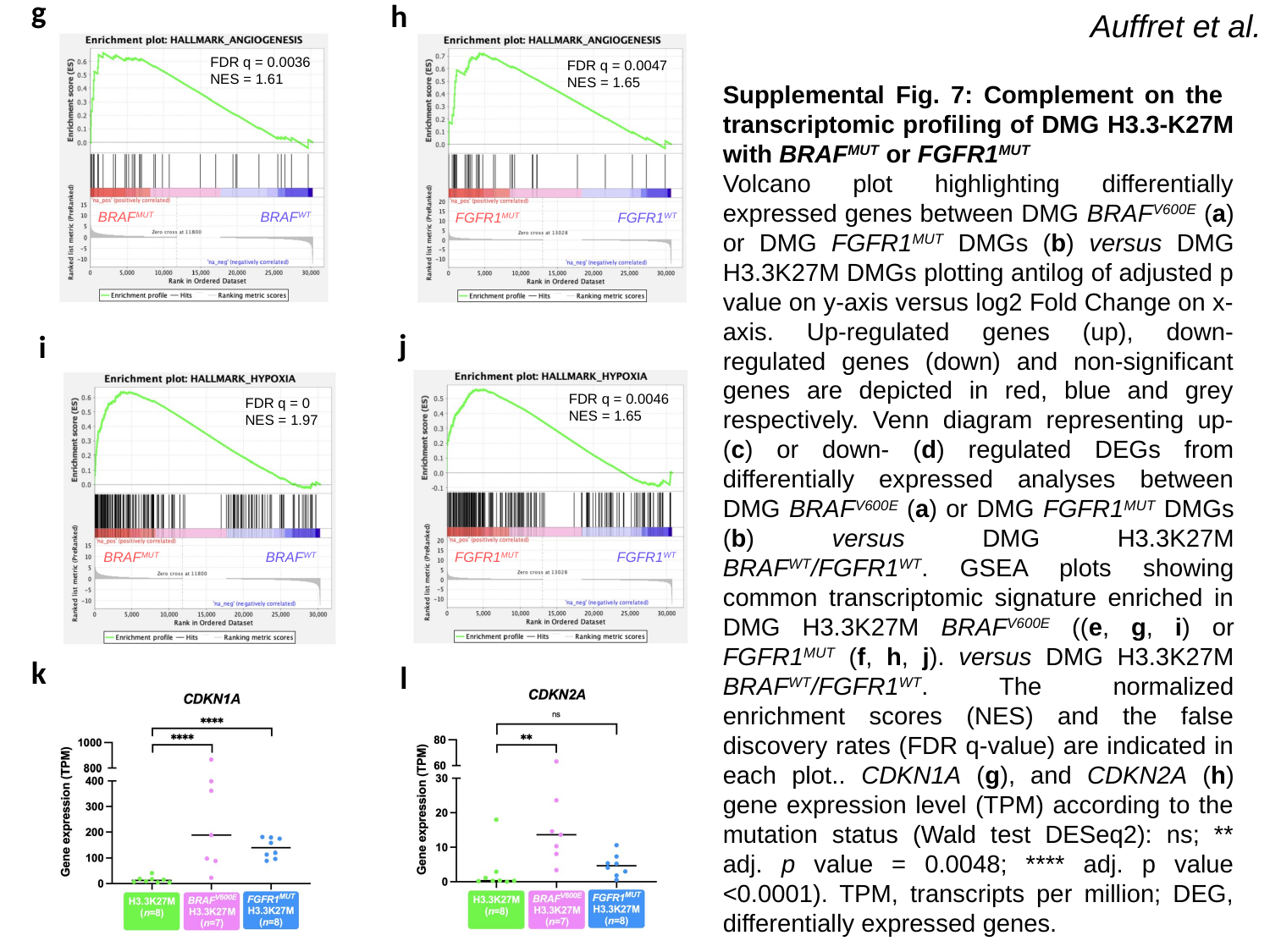

Auffret et al.
g
h
FDR q = 0.0036
NES = 1.61
FDR q = 0.0047
NES = 1.65
Supplemental Fig. 7: Complement on the transcriptomic profiling of DMG H3.3-K27M with BRAFMUT or FGFR1MUT
Volcano plot highlighting differentially expressed genes between DMG BRAFV600E (a) or DMG FGFR1MUT DMGs (b) versus DMG H3.3K27M DMGs plotting antilog of adjusted p value on y-axis versus log2 Fold Change on x-axis. Up-regulated genes (up), down-regulated genes (down) and non-significant genes are depicted in red, blue and grey respectively. Venn diagram representing up- (c) or down- (d) regulated DEGs from differentially expressed analyses between DMG BRAFV600E (a) or DMG FGFR1MUT DMGs (b) versus DMG H3.3K27M BRAFWT/FGFR1WT. GSEA plots showing common transcriptomic signature enriched in DMG H3.3K27M BRAFV600E ((e, g, i) or FGFR1MUT (f, h, j). versus DMG H3.3K27M BRAFWT/FGFR1WT. The normalized enrichment scores (NES) and the false discovery rates (FDR q-value) are indicated in each plot.. CDKN1A (g), and CDKN2A (h) gene expression level (TPM) according to the mutation status (Wald test DESeq2): ns; ** adj. p value = 0.0048; **** adj. p value <0.0001). TPM, transcripts per million; DEG, differentially expressed genes.
BRAFMUT
BRAFWT
FGFR1MUT
FGFR1WT
j
i
FDR q = 0.0046
NES = 1.65
FDR q = 0
NES = 1.97
FGFR1MUT
FGFR1WT
BRAFMUT
BRAFWT
k
l
